# Multiscale drainage dynamics with Haines jumps monitored by stroboscopic 4D X-ray microscopy

**DOI:** 10.1073/pnas.2305890120

**Published:** 2023-12-26

**Authors:** Kim Robert Tekseth, Fazel Mirzaei, Bratislav Lukic, Basab Chattopadhyay, Dag Werner Breiby

**Affiliations:** ^a^Department of Physics, Norwegian University of Science and Technology, 7491 Trondheim, Norway; ^b^ESRF - The European Synchrotron, Grenoble 38043, France; ^c^Department of Microsystems, University of South-Eastern Norway, 3184 Borre, Norway

**Keywords:** multiscale imaging, multiphase flow in porous media, interfacial dynamics, stroboscopic X-ray computed tomography

## Abstract

Slow multiphase flow in porous media is intriguing because its underlying dynamics is almost deterministic, yet depends on a hierarchy of spatiotemporal processes. There has been great progress in the experimental study of such multiphase flows, but three-dimensional (3D) microscopy methods probing the pore-scale fluid dynamics with millisecond resolution have been lacking. Yet, it is precisely at these length and time scales that the crucial pore-filling events known as Haines jumps take place. Here, we report four-dimensional (4D) (3D + time) observations of multiphase flow in a consolidated porous medium as captured in situ by stroboscopic X-ray micro-tomography. With a total duration of 6.5 s and 2 kHz frame rate, our experiments provide unprecedented access to the multiscale liquid dynamics. Our tomography strategy relies on the fact that Haines jumps, although irregularly spaced in time, are almost deterministic, and therefore repeatable during imbibition-drainage cycling. We studied the time-dependent flow pattern in a porous medium consisting of sintered glass shards. Exploiting the repeatability, we could combine the radiographic projections recorded under different angles during successive cycles into a 3D movie, allowing us to reconstruct pore-scale events, such as Haines jumps, with a spatiotemporal resolution that is two orders of magnitude higher than was hitherto possible. This high resolution allows us to explore the detailed interfacial dynamics during drainage, including fluid-front displacements and velocities. Our experimental approach opens the way to the study of fast, yet deterministic mesoscopic processes also other than flow in porous media.

## Significance

Multiphase flow in porous media is critical to industrial, biological, and environmental processes. The fluid dynamics exhibits a multitude of phenomena at different time and length scales, and because porous media are generally three-dimensional (3D) and opaque, it is challenging to measure the fast dynamics occurring inside the material. Haines jumps are sudden pore-filling events taking place on a millisecond timescale during drainage. We demonstrate that the multiphase flow dynamics, including Haines jumps, can be cyclically repeated and imaged in 3D movies using an original stroboscopic X-ray computed tomography technique. The unprecedented time resolution enables us to explore interfacial movements and velocities associated with fast fluid reconfigurations, opening new avenues for research in this societally important field.

The societal relevance of multiphase fluid flow, including drainage, in porous media is high (1–4) given both its ubiquitous presence in biology and geology in nature, as well as the technological applicability in various disciplines including CO_2_ storage in depleted petroleum reservoirs, soil irrigation, and gas diffusion layers in fuel cells. Multiphase fluid flow in porous media is a complex phenomenon, and while properties such as relative permeability and capillary pressure are commonly estimated on the macroscopic Darcy scale by assuming that pore-scale phenomena and inertial effects can be neglected, these assumptions are increasingly questioned (5–10).

At low saturation values, film and corner flow are known to contribute significantly to the hydraulic conductivity (11). At higher saturation values, capillary-dominated pore-scale flow dynamics exhibits a hierarchy of length- and timescales. On a time scale of seconds to hours relaxation following transient events reconfigures the fluid topology (12). For imbibition, the governing processes include piston-like fluid-front displacements and swelling of both wetting layers and menisci in corners may cause snap-off of the nonwetting phase. During drainage, the dynamics can be split into slow reversible fluid–fluid interfacial changes known as isons (13, 14) and fast irreversible pore-filling events within tens of milliseconds termed rheons (13, 14) or Haines jumps (15). In partially saturated samples, ganglions distributed throughout the porous medium can connect with the intruding nonwetting phase in coalescence events. These pore scale phenomena and their characteristic timescales are illustrated in *SI Appendix*, Fig. S1. While the fast millisecond-scale phenomena have been resolved in idealized two-dimensional (2D) samples, these phenomena have hitherto proven elusive in more realistic three-dimensional (3D) media owing to technical challenges. Porous 3D media inherently have increased connectivity and complexity as compared to their 2D counterparts, and developing techniques that can image even the fastest pore scale phenomena in four-dimensional (4D) (=3D + time) is crucial for assessing their influence on the macroscale properties.

Of the rapid phenomena mentioned, Haines jumps are arguably the most studied. Considerable efforts have been put forth to characterize these instabilities through pointwise measurements such as pressure readings and acoustic emissions (16–18). The statistics of pore-filling events associated with Haines jumps was first experimentally documented by Måløy et al. in a 2D Hele-Shaw cell through pressure readings (16, 19). Investigations of the dynamics of Haines jumps when air bubbles are pushed through a constricted capillary have demonstrated that in addition to the capillary effects arising from surface tension, also inertial effects can be present for certain fluid flow configurations, specifically with low Ohnesorge numbers (20). Through measurements of acoustic emissions associated with pore-scale fluid instabilities, DiCarlo et al. found that Haines jumps occur on a timescale of milliseconds for a water-air system in a sand-pack sample (17). Using a similar acoustic technique, Moebius et al. elaborated on the origin of these characteristic hydroacoustic emissions and linked them to Haines jumps, snap-off and liquid bridge rupture (18).

**Fig. 1.**
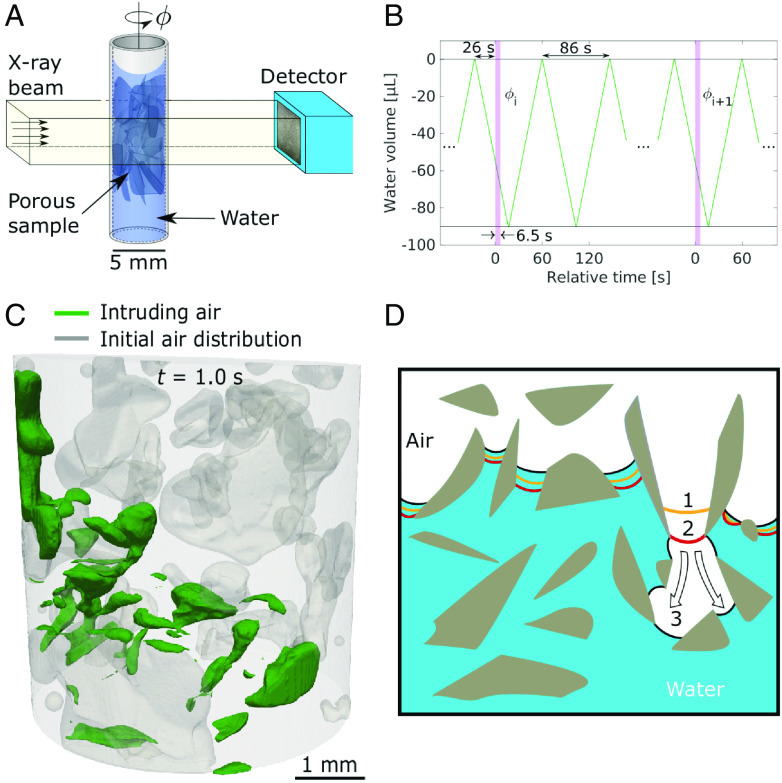
Experimental overview. (*A*) Illustration of the setup with the glass shard sample flooded with water. The water level was cycled to above the glass shards and thus above the camera FoV before the water extraction was initiated. (*B*) Water cycling and stroboscopic X-ray measurements. After draining for 26 s, the camera was triggered to capture 13,000 images at 2.0 kHz at projection angle $\phi_{i}$. Then, the data were stored, the sample rotated to the next projection angle $\phi_{i+1}$, and the camera prepared for the next triggering signal. (*C*) Reconstructed 3D perspective image of the intruding air phase 1.000 s into the drainage dynamics. (*D*) Illustration of a Haines jump, with three color-coded timesteps. Note how the neighboring water–air interfaces participate in the jump.

An important aspect of Haines jumps is their non-locality, as an extended region of the fluid-fluid interface tends to be affected (6, 13), cf. Fig. 1*D*. The nonlocality is often disregarded in drainage models such as invasion percolation (21) even though it is known to be an important feature of Haines jumps, as remarked by Armstrong and Berg (6, 22). In their studies, a 2D micromodel monitored by a high-speed camera was used to capture drainage dynamics, demonstrating the presence of nearby fluid–fluid interfaces retracting as an integral part of Haines jumps. Furthermore, they directly observed that the Haines jump dynamics typically lasts 5 to 30 ms, depending on the interfacial tension and the viscosity of the fluids. A 2D drainage study by Moebius and Or with typical pore diameters in the millimeter length scale (5) showed flow rate–dependent Haines jump interfacial velocities and the presence of meniscus oscillations indicative of inertial effects. Both studies reported the interfacial velocities to exceed the system average (Darcy) flow rate, by as much as three orders of magnitude (5, 6). Under such conditions, the distances that interfacial disturbances propagate during Haines jumps significantly exceed the length scale of the diffusive mixing of the fluids. Consequently, both spatial and temporal averaging must be applied to account for the energy dissipation of these events when upscaling from pore-scale to macroscale (9).

X-ray microcomputed tomography (µCT) has proven highly suitable for revealing internal structure and liquid dynamics in porous media (23, 24). With fourth generation synchrotron facilities and fast X-ray detector systems, high simultaneous spatial and temporal resolution can nowadays be obtained, allowing dynamic phenomena to be imaged with subsecond time resolution (25–27). In porous media, these advances have permitted pore-scale imbibition and drainage experiments such as capillary rise (28), snap-off (29), ganglion dynamics (10), and Haines jumps (7) to be studied. Recent developments within computational imaging have markedly improved the time resolution through reconstruction algorithms utilizing compressive sensing (30), machine learning (31), and a priori sample information (32, 33). However, all previously reported studies of fast pore-scale phenomena in 3D porous media have thus far only been able to capture the liquid configuration states before and after the abrupt liquid reconfigurations (7, 10, 34), with a time-resolution approaching 1 s in 3D reconstructions (35) and 40 ms in 2D radiographic projection images (36).

Here, we present a stroboscopic computed tomography technique that enables repeated pore-scale phenomena to be imaged in 4D with submillisecond temporal resolution. Consequently, the fluid dynamics could be studied in unprecedented detail, allowing quantitative information of the finer interfacial dynamics of these liquid instabilities to be extracted.

## Results

### Stroboscopic Imaging of Interfacial Dynamics.

**Fig. 2.**
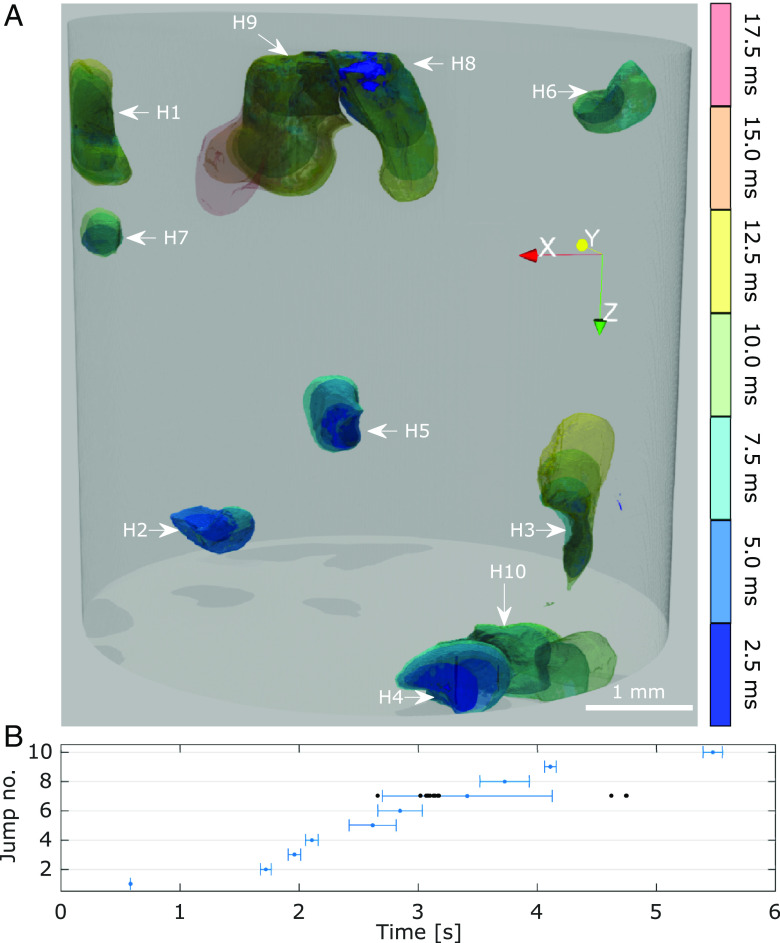
Visualization of Haines jumps H1 to H10. (*A*) Color-coded 4D rendering of the 10 observed Haines jumps. The coloring scheme indicates the temporal evolution of each jump individually, and the arrows indicate the starting location of the pore-filling events. The glass shards have not been rendered for readability. Gravity is along *z*. (*B*) Time indication for the onset of each jump, defined as the average temporal location from the 14 projections used in the reconstruction. The first jump was used as a synchronization timestamp, giving zero temporal deviation. The error bars for the subsequent jumps represent one SD of the temporal jump locations relative to the first jump. In 1 of the 14 time series, H7 actually occurred before H6, in three others H7 occurred after H8 and H9 (as indicated by the black dots, two are overlapping), hence giving the large error bar and illustrating the pseudo-repeatability.

Stroboscopic hard X-ray micro-computed tomography (4D-CT) as outlined in Fig. 1 was used to capture a unique 4D dataset of the drainage of a porous medium with a time resolution of 0.5 ms during 6.5 s, thus covering more than four decades of temporal dynamics. Similarly, three orders of magnitude in length (10 μm to 10 mm) were covered. The data enabled the detailed interfacial dynamics of drainage, including a multitude of Haines jumps, to be followed in 4D, providing insights into the dynamics of instabilities at the pore scale. With the submillisecond time resolution, we were able to monitor both the slower isons processes as reported in previous studies, and also the resolved rapid instabilities related to Haines jumps, in situ and in 4D, cf. Fig. 2.

The fundamental assumption underpinning our experiment is that the intruding nonwetting fluid (here: air) displacing the wetting (water) phase during repeated imbibition-drainage cycles exhibits the same flow pattern in each cycle, given a sufficiently low flow rate, cf. To promote repeatable behavior, we chose a strongly water-wet highly irregular 3D network of inert glass shards sintered in a glass capillary (cf. *SI Appendix*, *Complementary Results* and Fig. S2). The stroboscopic time series captured the fluid dynamics, including Haines jumps, as air displaced 0.5 M KI doped water, cf. Movie S1. A flow rate of 125 µL/min was chosen to ensure that several Haines jumps could be observed within the timeframe available, while maintaining a capillary-dominated flow regime. The corresponding Darcy velocity v was 0.1 mm/s, and the capillary number $Ca=\mu v/\sigma =1.4\cdot 10^{-6}$, where μ=0.96 mPa · s is the wetting phase viscosity and σ=72.5 mN/m the interfacial tension (37, 38). Furthermore, the Bond number was $Bo=\rho ga^{2}/\sigma =3.2\cdot 10^{-3}$, where ρ=1.059 g/cm^3^ is the doped water density (37), g=9.81 m/s^2^ is the gravitational acceleration and a=150 μm is a typical pore size. In total 30 stroboscopic radiographic 6.5 s time-series at a framerate of 2.0 kHz were measured, of which the first 14 were seen to exhibit a quasirepeated flow pattern, before the pattern changed into a slightly different, still repeated, mode. These 14 projection series were fed into the iterative prior image constrained compressive sensing (PICCS) (32) algorithm to obtain the 4D reconstructions. Here, we present a detailed analysis of the water–air interfacial dynamics, with special attention given to the Haines jumps.

A perspective 3D snapshot of the full field of view (FoV) with the intruding air phase 1.000 s into the dynamic measurements is presented in Fig. 1*C*, see also *SI Appendix* and Movie S2. Noteworthily, the intruding air phase was observed to merge with a trapped air ganglion (“bubble”) residing in the porous medium. Before this coalescence event, the trapped air pocket was seen to be contacted and slightly deformed by the intruding air phase, with only a thin water layer separating the advancing air front from the air bubble. When the water layer separating these menisci broke, the trapped air quickly rearranged by expanding at connected water–air interfaces, equilibrating the pressure, cf. *SI Appendix* and Movie S2. Noteworthily, the intruding air phase was observed to merge with a trapped air ganglion (“bubble”) residing in the porous medium. Before this coalescence event, the trapped air pocket was seen to be contacted and slightly deformed by the intruding air phase, with only a thin water layer separating the advancing air front from the air bubble. When the water layer separating these menisci broke, the trapped air quickly rearranged by expanding at connected water–air interfaces, equilibrating the pressure, cf. *SI Appendix*, Fig. S9.

In total 10 Haines jumps, labeled H1 to H10, were discerned during the 6.5 s of recorded dynamics. Reassuringly, these fast liquid reconfigurations could be directly observed in all the 14 projection series. A 3D rendering depicting the temporal evolution of the Haines jumps is presented in Fig. 2. To better grasp how these events are spatially connected, consider Movie S2. The Haines jumps developed in all Cartesian directions, including one jump (viz H3) in the opposite direction of gravity, consistent with capillary forces dominating the flow dynamics.

The first jump, H1, was used in the analysis as a timestamp for synchronizing the 14 timeseries and is thus assigned zero temporal spread, cf. Fig. 2*B*. The duration of the jumps varied from 10 to 20 ms with an average of 12.1 ± 4.4 ms, whereas the average waiting time between the instabilities was estimated to be 0.5 ± 0.4 s. The error bars in Fig. 2*B*, denoting one SD, reveal that the exact temporal onset of each jump varied slightly between each fluid cycle. A higher spread is observed for H7 as for three of the 14 cycles the order was temporarily shifted such that H7 occurred after H9, testifying to the nonexact repeatability.

### Interfacial Dynamics of a Single Haines Jump.

**Fig. 3.**
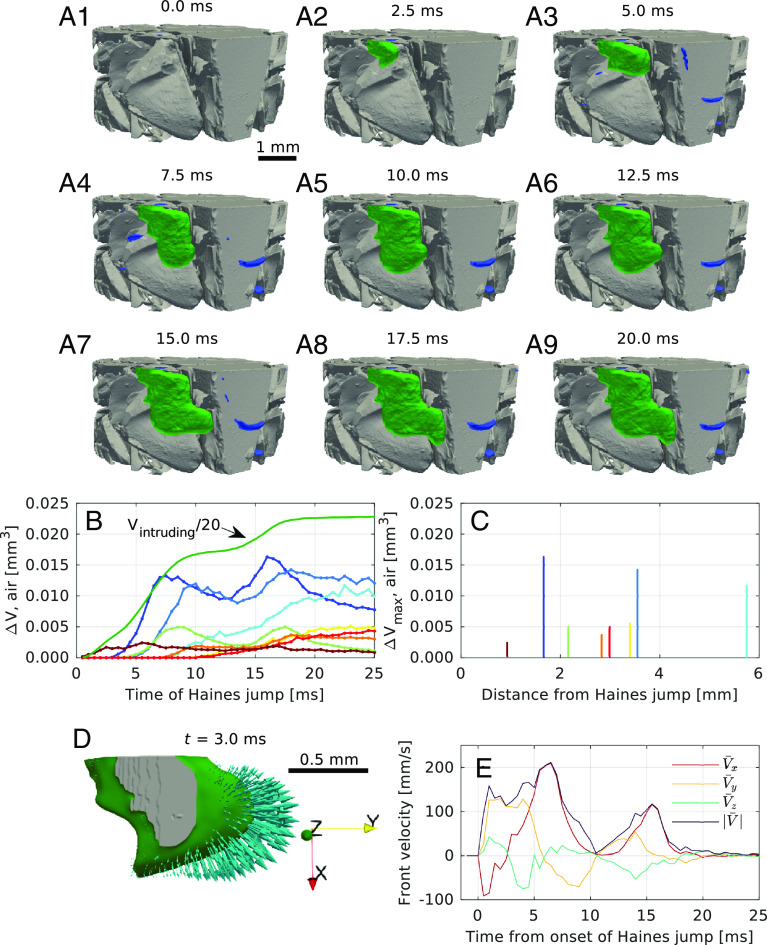
Interfacial dynamics of Haines jump H9, filling two pore bodies in a crevice located near the glass tube wall. (*A*1–*A*9) Selected 3D volume-of-interest renderings of the evolving Haines jump, lasting ~20 ms. Intruding and receding air volumes are painted green and blue, respectively, while the static glass shards are gray. The remaining volume, rendered transparent, was filled mainly with water and a few stationary air pockets. During the first 11 ms, the intruding air filled one pore body until it reached a narrow pore throat. The air then bounced back, headed into another direction, and filled a second pore before stabilizing. (*B*) Time-development of the invading air volume, and of the eight largest simultaneously retracting air volumes, showing clear oscillating behavior as the jump progressed. The color coding is arbitrary for each meniscus. (*C*) The same eight retracting air volumes as in (*B*), plotted as a function of distance from the Haines jump, exhibiting no correlation. (*D*) Visualization of the measured velocity field of the intruding air phase. (*E*) Average velocity components of the 10% largest velocity vectors, providing insight into the region of the interface that moves the most rapidly. Note that the velocity field was zero prior to the jump and that the field returned to a full standstill after the jump had finished.

Besides the location and temporal evolution of the observed Haines jumps drawn in Fig. 2, there is a wealth of quantitative kinetic information to be gained. Fig. 3 presents a detailed visualization of the advancing and retracting menisci associated with Haines jump H9 (Movie S3). Whereas most Haines jumps were seen to fill only one pore, H9 filled two large pores in quick succession and its invasion front changed direction three times over the course of about 20 ms as the air front explored its way through the rigid glass shard labyrinth. The reduction in water saturation within the FoV increased approximately linearly for the first 8 ms before it reached a plateau after about 11 ms, cf. Fig. 3*B*. The intruding air front was subsequently redirected and continued to move until it came to a temporary halt after approximately 20 ms. Inspecting the dynamics near the plateau reveals that the air–water meniscus bounced back, with a plausible explanation being inertia, as reported from 2D numerical micromodeling of Haines jumps (5). A discussion related to the oscillations and to the conservation of volumes during H9 can be found in *SI Appendix*, Fig. S17 and *Complementary Results*.

Fascinatingly, the neighboring air–liquid menisci can be seen to oscillate synchronously with the advancing pore-filling instability, as shown in Fig. 3*B* for H9. The maxima of the retracted interfaces occurred just before and after the intermediate halt of the Haines jump. Necessarily, the neighboring menisci started to move at later points in time, giving a time delay between the primary Haines jump and the dynamics of the neighboring retracting interfaces. Noteworthily, the distances from the Haines jump to the retracting menisci, both along Euclidean and tortuous distances, are only weakly correlated with the time delay, cf. *SI Appendix*, Fig. S16, suggesting a signal velocity of 0.33 m/s ($R^{2}=0.38$) for the tortuous path. While the weak correlation at first glance could seem surprising, recall that the liquid redistribution must equilibrate dynamically throughout the porous sample. It is thus not to be expected that a linear temporal relation should exist for the fluid equilibration between the pore bodies. Volumes of air that have pulled back as a function of distance from the pore-filling event (“jump”) are plotted in Fig. 3*C*, exhibiting no correlation. To the contrary, one of the largest retracted volumes within the FoV is seen to occur the farthest away possible (~6 mm) from the jump, demonstrating that the pressure variations of the jump influenced the air–water interface across the whole FoV. This independence of the dynamics from the distance to the jump suggests that major liquid-air reorganizations associated with the jump are likely to take place also outside of the FoV.

Knowing the spatiotemporal evolution of the air–water menisci, the interfacial velocity field can be extracted quantitatively, as shown for H9 in Fig. 3*D* and *SI Appendix*, Fig. S14. The estimated interfacial velocity vector fields for selected timesteps confirm that the velocity tended to be largest near the center of each meniscus. The average fluid front velocity and the Cartesian velocity components for the 10% highest velocities are plotted as function of time in Fig. 3*E*, arguably giving a reasonable estimate for the air invasion front velocity. Note that the interface movement came to a temporary halt after ∼10 ms. The total velocity before the halt exceeded the velocity after the halt by a factor of approximately 2. In lack of a better proxy for the momentum, note that the peak velocity, which approached a maximum of about 210 mm/s, suggests a local Reynolds number Re=vdρ/∼70 (with interfacial velocity v, pore diameter d∼ 300 µm, water density ρ = 1.059 g/cm^3^, and dynamic viscosity *µ* = 0.96 mPa · s). This high Re value indicates that inertial effects are significant. Noteworthily, as the fluids reached a stable configuration a significant rebound was observed (cf. *SI Appendix*, Fig. S17), giving further evidence that inertial effects were present during these rapid pore invasions (5). Modeling the rebound as an exponentially damped sine wave yields a damping ratio of 0.67 ± 0.03, consistent with an underdamped system. A similar rebound effect with distinct oscillations was observed for several of the Haines jumps in the current study (H1, H3, H8, H9), while nonoscillatory slight retractions towards the end of the jump were seen for H2, H4, H5, H6, and H10. Only for H7, a monotonically progressing filling of the pore space was observed. The details of the dynamics depend on the local pore geometry and are a topic for further study.

## Discussion

### On the Repeatability of Haines Jumps.

**Fig. 4.**
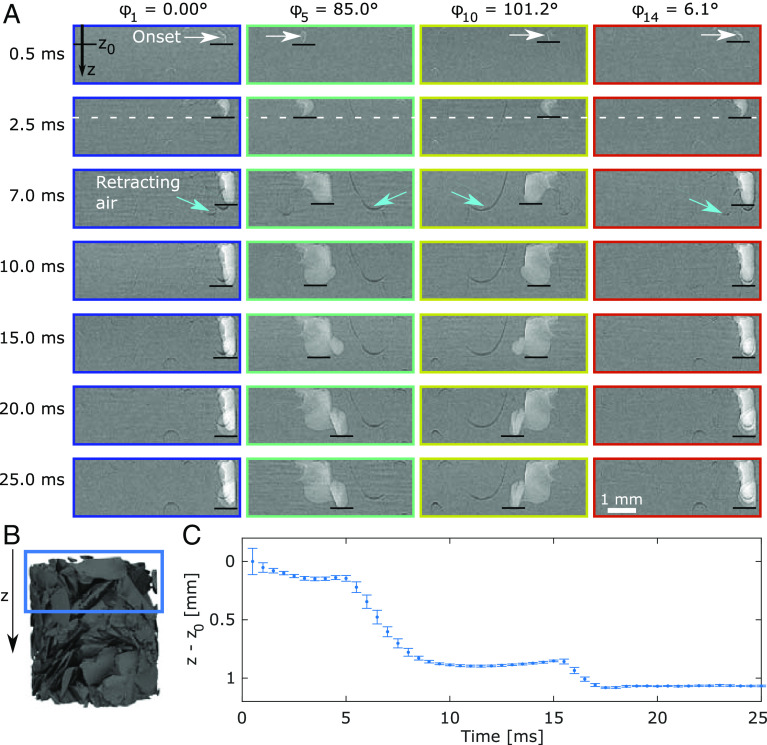
Repeatability of Haines jump H9. (*A*) Selected radiographs from four stroboscopic time series measured at projection angles $\phi_{i}$, zooming in on H9 in space and time. Corresponding radiographs obtained 50 ms prior to the onset of the jump were subtracted from each of these images to cancel the stationary structures, thereby highlighting the dynamics. Bright regions represent intruding air, with the white arrows indicating the onset of the jump in the top row. The small dark features from 7.0 ms onwards (teal arrows) are water-filled regions recently occupied with air which has retracted. The black horizontal lines indicate the farthest advanced point on the intruding air–water meniscus. (*B*) Region of interest used in (*A*). (*C*) Position of the farthest advanced point on the meniscus, averaged over the 14 projections, with the error bar signifying one SD. Larger deviations can be observed where the interfacial velocity was higher, i.e., around 7 and 16 ms.

The core assumption of our measurement strategy was that the drainage dynamics was a repeatable process, which necessarily includes the Haines jumps. Radiographs for four of the fourteen different projection angles as a function of time for H9 are shown in Fig. 4*A*, with the farthest progressed point on the water-air interface marked with a horizontal line. A remarkable similarity can be seen, as further emphasized in Fig. 4*C*, evidencing the repeatability of the liquid dynamics between the cycles, despite being separated in time by several hours. Essentially, this behavior validates the repeatability hypothesis underlying our experimental “hydraulic pump, X-ray probe” method.

Clearly, in a sufficiently simple and ideal classical system, the response to an identical stimulus should be deterministic and repeatable. In the present context of multiphase flow, the study of a capillary with a single constriction can serve as such an ideal system, yielding highly reproducible results (20, 39). Conversely, for nonlinear dynamic systems, the state evolves with time and popularly known as the butterfly effect, tiny variations in the initial and/or boundary conditions can lead to qualitatively different dynamics. For multiphase flow in a complex sample system like the one studied here, such small variations can be ascribed to many plausible influences: slight variations in external pressure, humidity, and temperature; radiation-induced local heating with consequences like pressure changes, increased evaporation and bubble formation; and X-ray radiation damage (cf. *SI Appendix*, *Experimental Considerations and Procedures*) in the form of chemical modifications changing the wetting properties and/or releasing impurities into the water, possibly with associated Marangoni effects caused by differences in surface tension (40)—to mention just the most likely mechanisms.

It was not a priori obvious that the multiphase flow that we studied would be sufficiently repeatable to render our analysis feasible. As Haines jumps are fast and delicate events spatially connected across large volumes, it is not granted that these millisecond events should be repeatable over numerous cycles spanning several hours in a complex porous environment exposed to an intense radiation field, as in our experiment. Considerable effort was consequently invested into the sample design, and sintered glass shards were chosen because of their irregular shapes (cf. *SI Appendix*, Fig. S2). The resulting highly angular pore spaces are realistic and lead to hydraulic conduction by corner flow (11). Moreover, the dissimilar pore spaces give a high likelihood of having just a few preferred pathways with comparatively low resistance to liquid flow through the sample. Note that the sharp protrusions of glass shards are also prone to provide pinning centers for the menisci, increasing the probability of both corner flow and sizable Haines jumps (41). For more regular samples, like glass bead-packs with essentially monodisperse beads, the pore volumes will be more similar, the pore throat distribution significantly narrower, and the number of energetically similar flow patterns through the sample increased, with a consequence being that repeated flow dynamics is harder to realize. As described, the onset of one of the Haines jumps, specifically H7, was observed to be somewhat erratic, cf. Fig. 2. We do not have sufficient spatial or chemical resolution to resolve why this Haines jump was less predictable, and the reason might be any one or a combination of the phenomena mentioned above.

To summarize, clearly depending on the properties of the porous network, but also on the nature of the fluids themselves like viscosity and interfacial tension, and on the flow conditions [e.g., Re-number (42)], there must be a transition between deterministic and random flow patterns. These considerations link directly to key concepts in contemporary theoretical models of multiphase flow in porous media, e.g., frameworks based on non-equilibrium thermodynamics and statistical mechanics (9, 43, 44), which might also provide links between the pore-level phenomena and the macroscopic observables. The present work demonstrates and discusses the effective repeatability of multiphase flow dynamics in porous media.

### Coalescence Event.

The coalescence of the trapped air ganglion with the intruding air phase has interesting implications for the imbibition part of the liquid cycling (which was not monitored). Because this coalescence event was observed in all the stroboscopic time series during drainage (*SI Appendix*, Fig. S9), there must be a corresponding break-up event taking place in the imbibition part of the experiment. As the angular glass shards are strongly water-wet, it is likely that this event is driven by water flow along crevices and corners causing a repeatable snap-off event to occur during liquid injection (11, 45, 46). A natural continuation of the current study would be to study liquid dynamics during imbibition, expecting phenomena including cooperative filling, corner flow, and snap-off events to be observed in similar detail.

### Nonlocality and Inertia of Haines Jumps.

Through detailed quantitative analysis of the Haines jumps, we have shown that the local velocity can exceed 200 mm/s, giving observable inertial effects that could be modeled as an underdamped oscillator. The observed velocity and the inertial effects are in close agreement with observations made by Moebius and Or who used a millimeter-sized 2D model system with water and air (5), but has not previously been experimentally observed in 3D systems due to the lack of sufficient spatiotemporal resolution. Our important direct observation of meniscus oscillations caused by inertial effects can potentially influence the pore-filling sequence and thus the distribution of fluids (8). Furthermore, the interfacial velocity attained by the Haines jumps suggest that time and spatial averages with appropriate representative elementary volumes (REV) must be used if the energy dissipation associated with these events is to be calculated (cf. *SI Appendix*, Fig. S18), for example in connection with up-scaling from the pore-scale to the macroscale (9).

The nonlocal effects of capillarity in porous media have been reported based on simulations and experiments with 2D micromodels to reach several hundred micrometers depending on the interfacial tension and the mobility ratio (6, 22). Our study using a 3D sample system, observed cooperative effects extending at least several millimeters, in fact spanning the entire system size. We note here that in this experiment the mobility ratio is lower than in the study by Armstrong et al. (22), expectedly increasing the zone-of-influence. Another explanation for this increased zone-of-influence might be attributed to the increased connectedness of 3D samples relative to 2D micromodels, and the wider pore-throat distributions in the glass shard sample. The knowledge of the zone-of-influence in 3D systems is of paramount importance when selecting REVs for numerical modeling to capture all the relevant pore-scale dynamics (22).

### Future Studies.

Extending the liquid pump—X-ray probe technique to larger volumes and naturally occurring porous geomaterials like sandstone, is a topic for further investigation, as is the application to both forced and spontaneous imbibition. In our study, we considered an idealized porous medium not because it is realistic, but because it provides a reference that will allow future research to assess the effect of surfactants, impurities, roughness, and varying pore geometry. Exploring system parameters such as interfacial tension, viscosity, and flow rate could give further insight into the fundamental properties of fluid dynamics in 3D porous media. In order to gain a full understanding of the dissipation of inertial energy, the spatial extent of nonlocal perturbations and the effect of the capillary number (22), and the role of momentum on flow (8), need to be investigated. The repeatability concept introduced in the *Discussion*, including the transition to turbulent flow, raises new questions that should be explored. In this study, the frame rate, FoV, and acquisition time were limited by the internal camera memory and the image saving time (28, 47, 48). With further advances in data transfer speeds and acquisition protocols, stroboscopic imaging techniques could permit more projections to be gathered in a shorter amount of time, thus improving the reconstructed spatiotemporal resolution and contrast, both directly by facilitating more data to be collected, and indirectly by effectively having a more stable system through reduced dead-time between measurements. Note that our technique should be applicable also to other fast, yet repeatable intermittent processes, such as particle rearrangements in dense colloidal systems under weak periodic shearing.

In conclusion, we have devised a new stroboscopic measurement strategy capable of producing 4D movies with unprecedented submillisecond temporal resolution, monitoring multiphase flow in porous media, including the interfacial dynamics of Haines jumps with their associated plethora of complexities. We have shown that the flow pattern for slow displacements can be repeated to high accuracy over many cycles. With an iterative reconstruction algorithm exploiting the static porous network as a priori knowledge, we were able to reconstruct the 4D fluid front dynamics based on only 14 projections. Our approach to fast 4D X-ray microscopy of liquid dynamics in porous media has provided measured numerical values of several key parameters, which could hitherto only be probed indirectly or estimated theoretically. These parameters include the liquid–air interfacial velocity, the damping ratio of the fluid front displacement oscillations, and the nonlocality of the liquid instabilities. The direct observation of phenomena such as nonlocality of Haines jumps, coalescence, and interface oscillations addresses the question how these effects can best be accounted for when upscaling from pore-scale to Darcy scale in two-phase flow. We expect that continued refinement of the multiscale liquid pump—X-ray probe method will become an important tool to further advance the understanding of the pore scale phenomena and their implications on the macroscopic permeability properties. In a wider context, our technique should also be applicable to other fast and repeatable mesoscale processes.

## Materials and Methods

### Sample.

The porous sample consisted of sintered borosilicate glass shards inside a 1-mm-thick borosilicate capillary tube of 5 mm inner diameter and 90 mm height. An inlet at the bottom of the capillary allowed liquid water to be alternatingly injected and retracted. The top end of the capillary was kept open to the surroundings. The sample was filled with 0.5 M potassium iodide (KI) doped water through spontaneous imbibition, yielding an initial water saturation of 78 ± 2%. The sample was mounted on a custom-built stage equipped with a gas-tight high-precision syringe (Hamilton Company) operated by a linear translation stage (Newport, MFA-PP stage, and NSC-200 motor controller). A pressure transducer (Honeywell, 26PCA) was mounted near the bottom inlet.

### Stroboscopic µCT: Acquisition.

The µCT measurements were conducted at the ESRF beamline ID19, equipped with a long wiggler (1.5 T B_max_). Beam configurations with a filtered pink beam having peak energy near 70 keV were used. For the static-conditions, high-resolution prior scan, a sample-detector distance of 440 mm was used, with a PCO Edge 5.5 sCMOS detector coupled to a 500-µm LuAG:Ce scintillator via a 2× objective. During the stroboscopic measurements, 90 µL of doped water was cyclically injected and withdrawn at a flow rate of 125 µL/min, giving a periodicity of approximately 86 s and a capillary-dominated flow regime. A PCO Dimax S7 detector with a 3× objective coupled to a 500-μm LuAG:Ce scintillator provided an effective pixel size of 3.66 × 3.66 µm^2^ and a framerate of 2.0 kHz for approximately 6.5 s of measurement time before the images had to be saved to a permanent storage device. The camera was triggered 26 s after starting a new drainage cycle (cf. Fig. 1) to only acquire images while water was drained within the FoV. After acquisition of a time series, the images were saved to disk, the sample rotated to the next projection angle, and the detector prepared for the next trigger signal. For selecting the projection angles a golden ratio scheme was used, allowing post-experiment decision on which projections to use for reconstruction (49). The sample was positioned 2.5 m from the detector to provide propagation-based phase contrast (50).

### Stroboscopic µCT: Reconstruction.

The high-resolution µCT prior scan of the porous sample static geometry was reconstructed with the filtered back-projection algorithm (51). The prior scan was segmented with a supervised watershed algorithm. The stroboscopic time-series of dynamic projections were flat-field corrected and phase-filtered before being registered to the prior scan. The dynamic datasets were reconstructed with the PICCS reconstruction algorithm (32, 52), which utilizes the prior scan and total variation (TV) regularization to enable reconstructions with few projections. The PICCS objective function was minimized with a nonlinear conjugate gradient algorithm and the projection operators were GPU-accelerated using the open-source ASTRA toolbox (53). The PICCS data fidelity term *λ* was set to 30, while the parameter α weighting the prior image information to new information was set to 0.5.

### Stroboscopic µCT: Analysis.

To suppress reconstruction artifacts associated with few-view tomography, we worked with the volume differences between the initial reference volume at the onset of the dynamics and the subsequent volumes, thereby highlighting the dynamically changing regions. The velocity vector fields of the air–water interface were estimated by first finding the surface normals and surface curvature, using open-source software (54). We then calculated the length from each point on the interface at time $t_{n}$ to the point on the surface at time $t_{n+1}$ along the surface normal, as an estimate to the distance traveled, effectively giving the velocity vector field of the advancing interface. See *SI Appendix*, *Experimental Considerations and Procedures* for full experimental details.

## Supplementary Material

Appendix 01 (PDF)

Movie S1. Visualization of the first stroboscopic radiographic projection series containing a total of 13000 images. The first image has been subtracted from the consecutive images in order to visualize the intruding (white regions) and retracting (darker regions) air phase.

Movie S2. Visualization of the displaced air occurring over a reconstructed time of 5.6 s with a temporal resolution of 200 ms. Green indicates the intruding air phase, whereas the gray phase indicates the initial air distribution.

Movie S3. Visualization of the interfacial dynamics during Haines jump H9 in the main text. Note how the intruding air is redirected three times throughout the dynamics and how it bounces back in the end.

## Data Availability

Segmented 4D reconstruction and the corresponding velocity field for the discussed Haines jump H9 are available at https://doi.org/10.5281/zenodo.10359845 (55).

## References

[1] P. Xu, A. P. Sasmito, A. S. Mujumdar, Heat and Mass Transfer in Drying of Porous Media (CRC Press, 2019).

[2] J. Feder, E. G. Flekkøy, A. Hansen, Physics of Flow in Porous Media (Cambridge University Press, 2022).

[3] S. J. Kowalski, Ed., Drying of Porous Materials (Springer, Netherlands, 2007).

[4] M. J. Blunt, Multiphase Flow in Permeable Media: A Pore Scale Perspective (Cambridge University Press, ed. 1, 2017).

[5] F. Moebius, D. Or, Interfacial jumps and pressure bursts during fluid displacement in interacting irregular capillaries. J. Colloid Interface Sci. **377**, 406–415 (2012). 22520212 10.1016/j.jcis.2012.03.070

[6] R. T. Armstrong, S. Berg, Interfacial velocities and capillary pressure gradients during Haines jumps. Phys. Rev. E **88**, 43010 (2013). 10.1103/PhysRevE.88.043010 24229279

[7] S. Berg , Real-time 3D imaging of Haines jumps in porous media flow. Proc. Natl. Acad. Sci. U.S.A. **110**, 3755–3759 (2013). 23431151 10.1073/pnas.1221373110 PMC3593852

[8] A. Ferrari, I. Lunati, Inertial effects during irreversible meniscus reconfiguration in angular pores. Adv. Water Resour. **74**, 1–13 (2014).

[9] J. E. McClure, S. Berg, R. T. Armstrong, Capillary fluctuations and energy dynamics for flow in porous media. Phys. Fluids **33**, 83323 (2021).

[10] M. Rücker , From connected pathway flow to ganglion dynamics. Geophys. Res. Lett. **42**, 3888–3894 (2015).

[11] M. Tuller, D. Or, Hydraulic conductivity of variably saturated porous media: Film and corner flow in angular pore space. Water Resour. Res. **37**, 1257–1276 (2001).

[12] S. Schlüter, S. Berg, T. Li, H. J. Vogel, D. Wildenschild, Time scales of relaxation dynamics during transient conditions in two-phase flow. Water Resour. Res. **53**, 4709–4724 (2017).

[13] N. R. Morrow, J. O. Szabo, Physics and thermodynamics of capillary action in porous media. Ind. Eng. Chem. **62**, 32–56 (1970).

[14] J. C. Melrose, Interfacial phenomena as related to oil recovery mechanisms. Can. J. Chem. Eng. **48**, 638–644 (1970).

[15] W. B. Haines, Studies in the physical properties of soil: V. The hysteresis effect in capillary properties, and the modes of moisture distribution associated therewith. J. Agric. Sci. **20**, 97–116 (1930).

[16] K. J. Måloy, L. Furuberg, J. Feder, T. Jossang, Dynamics of slow drainage in porous media. Phys. Rev. Lett. **68**, 2161–2164 (1992).

[17] D. A. DiCarlo, J. I. G. Cidoncha, C. Hickey, Acoustic measurements of pore-scale displacements. Geophys. Res. Lett. **30** (2003), 10.1029/2003GL017811.

[18] F. Moebius, D. Canone, D. Or, Characteristics of acoustic emissions induced by fluid front displacement in porous media. Water Resour. Res. **48**, 11507 (2012).

[19] L. Furuberg, K. J. Måløy, J. Feder, Intermittent behavior in slow drainage. Phys. Rev. E - Stat. Physics, Plasmas, Fluids, Relat. Interdiscip. Top. **53**, 966–977 (1996). 10.1103/physreve.53.966 9964331

[20] P. A. Gauglitz, C. J. Radke, Dynamics of Haines jumps for compressible bubbles in constricted capillaries. AIChE J. **35**, 230–240 (1989).

[21] D. Wilkinson, J. F. Willemsen, Invasion percolation: A new form of percolation theory. J. Phys. A Math. Gen **16**, 3365–3376 (1983).

[22] R. T. Armstrong, N. Evseev, D. Koroteev, S. Berg, Modeling the velocity field during Haines jumps in porous media. Adv. Water Resour. **77**, 57–68 (2015).

[23] V. Cnudde, M. N. Boone, High-resolution X-ray computed tomography in geosciences: A review of the current technology and applications. Earth-Sci. Rev. **123**, 1–17 (2013).

[24] D. Wildenschild, A. P. Sheppard, X-ray imaging and analysis techniques for quantifying pore-scale structure and processes in subsurface porous medium systems. Adv. Water Resour. **51**, 217–246 (2013).

[25] R. Mokso , GigaFRoST: The gigabit fast readout system for tomography. J. Synchrotron Radiat. **24**, 1250–1259 (2017). 29091068 10.1107/S1600577517013522 PMC5665295

[26] E. A. Chavez Panduro , Real time 3D observations of portland cement carbonation at CO_2_ storage conditions. Environ. Sci. Technol. **54**, 8323–8332 (2020). 32525672 10.1021/acs.est.0c00578 PMC7467647

[27] F. García-Moreno , Tomoscopy: Time-resolved tomography for dynamic processes in materials. Adv. Mater. **33**, 2104659 (2021). 10.1002/adma.202104659 PMC11468671 34558111

[28] A. Piovesan , 4D synchrotron microtomography and pore-network modelling for direct in situ capillary flow visualization in 3D printed microfluidic channels. Lab Chip **20**, 2403–2411 (2020). 32514512 10.1039/d0lc00227e

[29] K. Singh , Dynamics of snap-off and pore-filling events during two-phase fluid flow in permeable media. Sci. Rep. **7**, 1–13 (2017). 28701699 10.1038/s41598-017-05204-4 PMC5507864

[30] E. Y. Sidky, X. Pan, Image reconstruction in circular cone-beam computed tomography by constrained, total-variation minimization. Phys. Med. Biol. **53**, 4777–4807 (2008). 18701771 10.1088/0031-9155/53/17/021 PMC2630711

[31] K. H. Jin, M. T. McCann, E. Froustey, M. Unser, Deep convolutional neural network for inverse problems in imaging. IEEE Trans. Image Process. **26**, 4509–4522 (2017). 28641250 10.1109/TIP.2017.2713099

[32] G. H. Chen, J. Tang, S. Leng, Prior image constrained compressed sensing (PICCS): A method to accurately reconstruct dynamic CT images from highly undersampled projection data sets. Med. Phys. **35**, 660–663 (2008). 18383687 10.1118/1.2836423 PMC2655145

[33] K. J. Batenburg, J. Sijbers, DART: A practical reconstruction algorithm for discrete tomography. IEEE Trans. Image Process. **20**, 2542–2553 (2011). 21435983 10.1109/TIP.2011.2131661

[34] T. Bultreys , Real-time visualization of Haines jumps in sandstone with laboratory-based microcomputed tomography. Water Resour. Res. **51**, 8668–8676 (2015).

[35] K. Singh , The role of local instabilities in fluid invasion into permeable media. Sci. Rep. **7**, 444 (2017). 28348395 10.1038/s41598-017-00191-y PMC5427855

[36] R. T. Armstrong , Subsecond pore-scale displacement processes and relaxation dynamics in multiphase flow. Water Resour. Res. **50**, 9162–9176 (2014). 25745271 10.1002/2014WR015858 PMC4328147

[37] J. R. Rumble, CRC Handbook of Chemistry and Physics (CRC Press, 2014), 10.1201/b17118.

[38] K. Ali, A. ul H. A. Shah, S. Bilal, A. ul H. A. Shah, Surface tensions and thermodynamic parameters of surface formation of aqueous salt solutions: III. Aqueous solution of KCl, KBr and KI. Colloids Surf. A Physicochem. Eng. Asp. **337**, 194–199 (2009).

[39] Z. Sun, J. C. Santamarina, Haines jumps: Pore scale mechanisms. Phys. Rev. E **100**, 023115 (2019). 31574718 10.1103/PhysRevE.100.023115

[40] Y. Edery, D. Weitz, S. Berg, Surfactant variations in porous media localize capillary instabilities during Haines jumps. Phys. Rev. Lett. **120**, 028005 (2018). 29376702 10.1103/PhysRevLett.120.028005

[41] R. Holtzman, M. Dentz, R. Planet, J. Ortín, The origin of hysteresis and memory of two-phase flow in disordered media. Commun. Phys. **3**, 1–7 (2020).

[42] B. D. Wood, X. He, S. V. Apte, Modeling turbulent flows in porous media. Annu. Rev. Fluid Mech. **52**, 171–203 (2020).

[43] A. Hansen, E. G. Flekkøy, S. Sinha, A. Slotte, A statistical mechanics framework for immiscible and incompressible two-phase flow in porous media. Adv. Water Resour. **171**, 104336 (2023).

[44] K. J. Måløy, M. Moura, A. Hansen, E. G. Flekkøy, R. Toussaint, Burst dynamics, upscaling and dissipation of slow drainage in porous media. Front. Phys. **9**, 796019 (2021).

[45] A. S. Ambekar, S. Mondal, V. V. Buwa, Pore-resolved volume-of-fluid simulations of two-phase flow in porous media: Pore-scale flow mechanisms and regime map. Phys. Fluids **33**, 102119 (2021).

[46] W. Lei, X. Lu, F. Liu, M. Wang, Non-monotonic wettability effects on displacement in heterogeneous porous media. J. Fluid Mech. **942**, 5 (2022).

[47] S. M. Walker , In vivo time-resolved microtomography reveals the mechanics of the blowfly flight motor. PLoS Biol. **12**, 1001823 (2014). 10.1371/journal.pbio.1001823 PMC3965381 24667677

[48] R. Mokso , Four-dimensional in vivo X-ray microscopy with projection-guided gating. Sci. Rep. **5**, 8727 (2015). 25762080 10.1038/srep08727 PMC4356984

[49] T. Köhler, “A projection access scheme for iterative reconstruction based on the golden section” in IEEE Nuclear Science Symposium Conference Record, J. A. Seibert, Eds. (IEEE, 2004), pp. 3961–3965.

[50] D. Paganin, S. C. Mayo, T. E. Gureyev, P. R. Miller, S. W. Wilkins, Simultaneous phase and amplitude extraction from a single defocused image of a homogeneous object. J. Microsc. **206**, 33–40 (2002). 12000561 10.1046/j.1365-2818.2002.01010.x

[51] A. C. Kak, M. Slaney, *Principles of Computerized Tomographic Imaging* (Society for Industrial and Applied Mathematics, 2001), 10.1137/1.9780898719277.

[52] P. T. Lauzier, J. Tang, G. H. Chen, Prior image constrained compressed sensing: Implementation and performance evaluation. Med. Phys. **39**, 66–80 (2012). 22225276 10.1118/1.3666946 PMC3257752

[53] W. van Aarle , The ASTRA Toolbox: A platform for advanced algorithm development in electron tomography. Ultramicroscopy **157**, 35–47 (2015). 26057688 10.1016/j.ultramic.2015.05.002

[54] A. AlRatrout, A. Q. Raeini, B. Bijeljic, M. J. Blunt, Automatic measurement of contact angle in pore-space images. Adv. Water Resour. **109**, 158–169 (2017).

[55] K. R. Tekseth, D. W. Breiby, Multiscale drainage dynamics with Haines jumps monitored by stroboscopic 4D X-ray microscopy. Zenodo. 10.5281/zenodo.10359845. Deposited 11 December 2023. PMC10769832 38147554

