# Supplementary material for: Multiscale drainage dynamics with Haines jumps monitored by stroboscopic 4D X-ray microscopy
Source: Proc Natl Acad Sci U S A. 2023 Dec 26;121(1):e2305890120. doi: 10.1073/pnas.2305890120 (PMC10769832; doi:10.1073/pnas.2305890120)
Supplement: Supplementary file 1 — Appendix 01 (PDF) [file pnas.2305890120.sapp.pdf]

PNAS

Supporting Information for
Multiscale drainage dynamics with Haines jumps monitored by stroboscopic 4D X-ray microscopy

Kim Robert Tekseth[1], Fazel Mirzaei[1], Bratislav Lukic[2], Basab Chattopadhyay[1], Dag Werner Breiby[1,3]
[1]Department of Physics, Norwegian University of Science and Technology (NTNU), Høgskoleringen 5, 7491 Trondheim, Norway
[2]ESRF, 71 Avenue des Martyrs, 38000 Grenoble, France
[3]Department of Microsystems, University of South-Eastern Norway (USN), 3184 Borre, Norway

*Corresponding author: Dag W. Breiby
Email: dag.breiby@ntnu.no

This PDF file includes:

Supporting text:
SI Appendix 1. Experimental considerations and procedures
SI Appendix 2. Complementary results

Figures S1 to S18:
Fig. S1. Spatiotemporal scales of capillarity
Fig. S2. Static 3D structure of the sintered glass shard sample
Fig. S3. Check for radiation damage
Fig. S4. Segmentation of the high-quality a priori CT data
Fig. S5. Difference of volumes highlights dynamics
Fig. S6. Estimating the velocity field of the intruding meniscus
Fig. S7. Pressure history throughout the experiment
Fig. S8. Pressure readings averaged over the cycles
Fig. S9. Coalescence between the intruding air and a ganglion bubble
Fig. S10. Stroboscopic projection time series for Haines jump H1
Fig. S11. 4D air invasion dynamics into the porous sample
Fig. S12. Estimate of advancing and receding contact angles
Fig. S13. Repeatability of Haines jump H9
Fig. S14. Interfacial velocity fields for the advancing air-water meniscus
Fig. S15. Volume balance during Haines jump H9
Fig. S16. Instability delay across zone of influence
Fig. S17. Mathematical fit of meniscus rebound oscillations
Fig. S18. Displacement as function of time during Haines jumps.

Legends for Movies S1 to S3
SI References

Other supporting materials for this manuscript include the following:

Movies S1 to S3

SI Appendix 1. Experimental considerations and procedures

Capillary-dominated porous media flow phenomena

The pore scale phenomena mentioned in the main text and their characteristic timescales are sketched in Fig. S1. Although many of these phenomena have been imaged using 2D micro-models and optical microscopy, none of the faster (i.e., milliseconds) phenomena have been measured previously in 4D (=3D + *time*).

Sample and sample stage

The sample system consisted of borosilicate glass shards sintered inside a borosilicate capillary tube. By sintering the glass shards, we ensured a static and inert consolidated porous network as a playground for the fluid dynamics. A high-quality CT scan of the consolidated porous sample was acquired before the liquid flow dynamics experiments, effectively serving as *a priori* information for the reconstruction algorithm. An inlet at the bottom of the sample allowed liquid water to be alternatingly injected and retracted, while the top end of the capillary was kept open to the surroundings. The sample was filled with 0.5 M potassium iodide (KI) doped water through spontaneous imbibition, resulting in an initial water saturation of 78±2% because of pockets of trapped air in-between the glass shards. Doped water provides enhanced absorption contrast relative to the air phase. The 0.5 M KI doped water has a density of $\rho = 1.059$ g/cm^3 (1), a dynamic viscosity of $\mu = 0.96$ mPa ·s (1), and a doped water-air surface tension of $\sigma = 72.5$ mN m^{-1} (2).

The sample was mounted on a custom-built sample stage equipped with a syringe pump. The syringe pump consisted of a Newport MFA-PP translational stage combined with an NSC-200 motor controller in which the smallest motor increment, termed a micro-step, translates 7.57 nm. With the given flow rate of 125 μL/min, there are 7680 micro-steps/s, approximately 3.84 steps per radiographic frame acquired during the stroboscopic measurements. The acceleration ramp-up was set to 153600 micro-steps/s^2. As exemplified in Supplementary Movie 1, this pump ensured smooth flow dynamics during the experiments. The syringe pump was outfitted with a gas-tight high-precision syringe (Hamilton Company). By attaching the syringe pump to the sample stage, one avoids disturbing the liquid supply lines during sample rotation. Furthermore, the liquid supply lines were made of high modulus polytetrafluoroethylene (PTFE) tubes to reduce volume changes of the tubes caused by pressure differences during flow (3). A pressure transducer (Honeywell, 26PCA) was mounted at the inlet of the sample capillary for pressure readings of the combined hydrostatic and capillary pressure. The sample stage was outfitted with a slip-ring (SenRing Electronics Co., China) allowing continuous unidirectional rotation without the need for rewinding.

Experimental setup and measurement strategy

The key principle of our study on drainage in a porous medium is to exploit the repeatability of the drainage dynamics to record time series at different projection angles, while cyclically injecting and withdrawing a fixed amount of water in the system. During the analysis, data from different events are then combined to form the impression of a single event represented in 4D. Thus, during the dynamic measurements, 90 μL of doped water was cyclically injected and withdrawn at a flow rate of 125 μL/min, giving a periodicity of approximately 86 s. This flow rate results in a capillary number $\mathrm{Ca} = \mu_i v_i / \sigma = 1.4 \cdot 10^{-6}$,

a viscosity ratio $\mathrm{M} = \mu_i/\mu_d = 2 \cdot 10^{-2}$, and a Bond number $\mathrm{Bo} = \Delta\rho g a^2/\sigma = 3.2 \cdot 10^{-3}$, yielding a capillary dominated flow regime with minor gravitational effects (4, 5). Here, $v = 0.11$ mm/s is the Darcy velocity and $a = 150$ μm is a typical pore size.

The μ-CT measurements were conducted at the European Synchrotron Research Facility beamline ID19 (Grenoble, France), optimized for micro-tomography studies at both high spatial and high temporal resolution. The beamline is equipped with a long wiggler (1.5T B_{max}) with a tunable gap providing a broad X-ray spectrum in white beam mode.

High-resolution scan

For the high-resolution scan (cf. Fig. S2) of the static porous structure prior to the fluid dynamics acquisitions, a filtered pink beam peaked near 65 keV was used. The beam was filtered with 2.8 mm of aluminum, 1.4 mm copper and 0.06 mm tungsten. The sample-detector propagation distance was 440 mm. The *a priori* scan of the static porous matrix was recorded using a PCO Edge 5.5 sCMOS camera optically coupled to a Hasselblad tandem microscope (210:100) with a 500 µm LuAG:Ce scintillator. The detector arrangement yielded 2560×2160 pixels per radiograph with an effective pixel size of 3.25×3.25 μm^2 using an objective lens with 2× magnification. 2000 projections from 0 to 180 degrees were captured with an exposure time of 50 ms each.

Dynamic scans

The fast dynamic acquisitions were performed after increasing the magnetic field and reducing the wiggler gap, which provides a harder X-ray spectrum with a considerable increase of flux with a peak around 70 keV, allowing more photons to be transmitted through the sample. The pink beam in this configuration was filtered with 2.8 mm aluminum, 0.34 mm copper and 0.06 mm tungsten. Given the energy shift caused by the reduction of the wiggler gap (i.e., higher energy spectrum) the resulting pink beam was of 70 kV and thus providing comparable contrast to the a priori static scan. The sample was positioned 2.5 m away from the detector to provide propagation-based phase contrast. A PCO Dimax S7 camera configured with a 1440×1328 pixels region of interest and a 3× magnification Hasselblad tandem microscope (300:100) coupled to a 500 µm LuAG:Ce scintillator provided an effective pixel size of 3.66×3.66 μm^2 and a framerate of 2000 Hz for approximately 6.5 s of total measurement time before the images had to be saved to a permanent storage device.

Due to the limited recording time of the camera, a triggering system was devised to only acquire images while the majority of the bulk water was drained within the field of view. The triggering signal was sent to the camera 26 s after the onset of a new drainage cycle. After acquisition of the stroboscopic images approximately 8 minutes of deadtime followed while images were saved to a permanent disk. During this deadtime the water was continuously cycled. After the radiographic series had been saved, the sample was repositioned to a new projection angle and the camera was put into triggering mode waiting for the next drainage time series to be recorded. This procedure was repeated 30 times, acquiring a total of ~390,000 images over a period of 3.2 hours.

For the projection angles we used an angular increment based on the golden ratio acquisition scheme,

$$\phi_{i+1} = \phi_i + \frac{\sqrt{5}-1}{2} 180° \approx \phi_i + 111.25° ,$$

which ensures that the most angularly unique (i.e., complementary) information, as compared to the already measured data, was recorded in every acquisition (6). The primary advantage of this acquisition scheme is that the selection of consecutive projections to be used in the reconstruction algorithm can be decided post experiment.

Radiation damage

The 4th generation extremely brilliant source at ESRF can easily cause radiation damage to samples. In the case of glass, this can cause darkening and structural changes, whereas vapor bubbles can be formed in the water (7–11). In our study, no structural changes of the glass sample nor formation of radiation-induced vapor bubbles could be observed by visual inspection of the first and last (i.e., 30th) projection series, as demonstrated in Fig. S3.

Our image acquisition protocol utilized a prospective pressure-gated acquisition scheme with reduced radiation dose compared to the similar retrospective acquisition scheme that continuously exposes the sample to radiation (12, 13). Still, radiation damage is inevitably a concern when using extremely brilliant 4th generation synchrotrons. First, irradiation of silicates is known to cause both darkening of the glass due to the creation of color centers, and compaction (7, 8). While a darkening of the borosilicate glass was observed, no structural changes in the capillary walls between the first and last projection series could be measured. Second, absorption of X-ray radiation can cause formation of vapor bubbles specifically and vaporization in general (9–11). We report no formation of vapor bubbles throughout the experiment within the FoV for the flow rate used in this study. However, we note that in complementary tests conducted with lower flow rates (<~60 µL/min) and smaller cycling volumes several vapor bubbles were seen to form and grow, and eventually get trapped. Evaporation, both natural and radiation-induced, can cause a net loss of fluid in each cycle, and thus be one explanation why the flow pattern was observed to slightly change after the first 14 stroboscopic projection time-series. Third, radiation induced changes in the wetting behavior might also influence the dynamics. Fourth, absorbed energy from the X-ray beam can cause sample heating, slightly modifying the relative volumes of the phases.

Image processing

Reconstructed raw and segmented cross sections of the artificial porous sample are provided in Fig. S4. Albeit demanding because of the low contrast, using the watershed algorithm, we could separate glass from doped water. By counting voxels excluding glass, we estimate a pore volume of 41±3 µL within the field of view, giving a macro-porosity of approximately 28±2%. Similarly, by counting air voxels we found an initial air saturation of 22±2%.

Reconstructed slices at two different timesteps are provided in Fig. S5, showing a reconstructed slice at $t = t_0$ at the onset of the jump H9 and the same slice at a later time $t = t_1$. Calculating the volume difference highlights the changes between the two timesteps, revealing a region with intruding air and a smaller volume of retracting air.

In the segmented tomograms, the interfaces were smoothed using open-source software (14). Fig. S6a exemplifies a reconstructed volume with an intruding air finger,

with clear voxel artefacts present. The interface was smoothed with both Gaussian and curvature smoothing operations as exemplified in Fig. S6b (14). Finally, surface normals were computed and projected onto the surface of the next timestep to describe the movement of the interface as shown in Fig. S6c. This displaced interface length divided by the time resolution provides an estimate for the interfacial velocities.

Pressure readings

Pressure readings with a logging frequency of 10 Hz and a resolution of 2.5 Hz were acquired throughout the experiment. Calibrated pressure readings obtained throughout the experiment are presented in Fig. S7, with the vertical lines indicating the temporal location of the 30 stroboscopic projection series. The figure also shows the pressure-volume curves obtained from the syringe pump and the pressure readings, demonstrating the expected presence of hysteresis in the system. These data also provide additional evidence for the repeatability of the liquid flow pattern between iterations. After the acquisition of the 14th radiographic projection series, the fluid displacement path slightly changed. Certainly, most of the Haines jumps could still be observed, however, they were appearing at different relative points in time. The 16 remaining projection series were excluded for the reconstruction as the neighboring air-water interface configurations were also different.

A closer look at the pressure curves during the stroboscopic acquisitions as a function of time is presented in Fig. S8, where the averaged pressure readings over the cycles are plotted. The temporal resolution of the pressure readings is clearly insufficient for correlating the pressure with the Haines jumps. The broad pressure increase occurring about 1 s into the measurement series is unambiguously associated with the coalescence of the bulk air phase with a trapped air bubble (ganglion). The limited frequency of the datalogger renders quantitative analysis of the pressure data difficult.

SI Appendix 2. Complementary results

The glass-shard porous medium and its limited fluid flow paths

A 3D rendering of the porous glass shard sample used in this study is given in Fig. S2. Although packs of spherical glass beads are often used in idealized drainage and imbibition experiments, we chose to use a sample consisting of irregular glass shards for mainly two reasons. First, as Fig. S2 demonstrates, the sample consisted of a wide range of pore body radii and throat radii, stemming from the large variability in shape and size of the glass shards. As such, the model sample is a close representation of the heterogeneously distributed pore space that is present in a wide range of natural materials (15). The sharp edges are also likely to act as pinning points for the water-air menisci. Second, the wide range of pore throat radii promotes a preferred flow pattern through the widest pores, consistent with flow regimes dominated by capillary effects. This preferred flow path is determined largely by the higher capillary pressure thresholds associated with narrower pore throats.

Coalescence between bulk-air with trapped air bubble

An important event observed during the drainage was the coalescence of the intruding air phase with a large air bubble (ganglion). After Haines jump H1, the air front continued developing towards an air ganglion. Before the coalescence event, only a thin water layer separated the two air-phases, ultimately leading to the breaking of the water layer and causing an immediate expansion of volume filled by air, see Fig. S9. The advancing air front is apparently compressing the ganglion, causing it to expand as it coalesces with the bulk air. After the initial expansion, the reconnected air-bubble was observed to rapidly fill two pore bodies within 20 ms, as marked with white arrows in Fig. S9a4. Correspondingly, the volume changes reached 0.6 mm^3 after 20 ms. The coalescence event occurred 1.239 s into the stroboscopic timeframe of 6.5 s, see Supplementary Movie S1.

Stroboscopic projection series reveals fast repeatable dynamics

Here, we further expand on the repeatability of the flow dynamics by presenting radiographic images of selected timesteps for all 14 stroboscopic projection series of H1, see Fig. S10. A reference image 50 ms before the event was subtracted from the displayed projections. The projection series have been sorted according to the measured projection angles ϕ_i. Haines jump H1 is characterized by its three-fingered structure, which can be seen to evolve in all 14 stroboscopic projection series.

Total displaced volume

Fig. S11a shows the displaced water from the onset of jump 1 until the completion of jump 10. In addition, the initial air saturation prior to the dynamics is visualized as semi-transparent gray. As some of the displaced volumes are disconnected, it is illustrative to also visualize how they are connected through the initial air saturation that remained stationary throughout the dynamics. Furthermore, in Fig. S11b we see the development of water displaced by air, before and after each jump, measured from the start of the first jump until the end of the last jump. The total amount of displaced volume within the field of view is 3 µL. In contrast, the total amount withdrawn by the pump is 12 µL, four times more than the displaced volume.

Estimate of contact angles and hysteresis through radiographic projections

An estimate of the contact angle and the associated hysteresis were obtained through radiographic projections during Haines jump H1 of a neighboring contracting water-air interface, cf. Fig. S12. Prior to the jump, the measured contact angle corresponds to the receding contact angle. As the jump progress, the neighboring meniscus locally imbibes and thus correspond to an advancing contact angle. The liquid-air and the liquid-solid vectors are approximately orthogonal to the beam direction. Through this analysis, an advancing/receding contact angle of 29°/20° was obtained, with a hysteresis of 9°. Although these contact angles are only approximate values, it ascertains that the glass shards are strongly water-wet.

More on the repeatability of Haines jump H9

The repeatability of the Haines jump H9 discussed in the main article (Fig. 4) is further evidenced in Fig. S13, here for the same angular orientation $\phi_0 = 111.24°$ obtained at two different cycles. A reference projection image acquired 50 ms before the onset of the Haines jump have been subtracted from the presented projections to highlight the changes, showing that the fluid was practically stationary prior to the jump. The first two columns show projections obtained from two cycles separated by 4 periods, while the third column shows the image differences between the two first columns. A slight discrepancy can be observed at the onset of the second part of the jump as shown in the difference image at 17.5 ms. This is the location where the fluid was moving the fastest, thus giving the largest discrepancies. The fluid came to rest at similar interfacial positions.

Quantitative analysis of Haines jump H9

Velocity estimate

The interfacial velocity field throughout the dynamics of Haines jump H9 is presented in Fig. S14a1-a9. Figure S14b shows the average velocity for the entire interface, whereas Fig. S14c shows the velocity field of the 10% highest velocity vectors, which is more indicative of the speed at which these interfaces travel.

Conservation of volume

Haines jumps are known to occur without changing the saturation of the present fluid phases. Instead, the fluid-fluid interfaces reorganize themselves as neighboring menisci retract in order to facilitate the displacement. Here we analyze the change in air volume during Haines jump H9. The volume of air having invaded the main pore and the simultaneous retraction of air from neighboring pores during H9 is presented in Fig. S15. The volume displaced by air in the invaded pore during the ~25 ms jump is approximately 0.45 mm^3. At other locations within the FoV, the directly observed volume of retracting air is about 0.04 mm^3, while the water volume withdrawn by the pump during the same time is about 0.05 mm^3, totaling 0.09 mm^3. One might argue that the summed volumes of the extracted water and air retraction should stay equal to the advancing air phase volume through the conservation of volume. However, the air in the sample will compress or expand with changes in pressure associated with the Haines jumps. The pressure readings (see SI Appendix 1) were not sufficiently precise to allow the compressibility of air to be taken quantitatively into account. The intruding and retracting curves in Fig. S15 are highly

correlated, having a correlation coefficient of 0.97. This apparent volume retraction might be taken as indirect evidence that the air-water interfaces in the neighboring pores have redistributed in order to facilitate the jump. Our primary explanation for the volume deviance is that liquid redistribution (i.e., air retraction) also outside of the detector FoV takes place. In fact, the vertical FoV of 4.8 mm accounts for approximately 10% of the overall height of the porous glass shard sample. Thus, assuming a significant amount of air retraction also outside the FoV helps accounting for the remaining air phase expansion during the Haines jump.

Signal velocity
As expected by causality, we note that the neighboring menisci start to move at later points in time, giving the time delay between the Haines jump and the dynamics of the neighboring retracting interfaces. Noteworthily, the distances from the Haines jump to the retracting menisci, both along Euclidean and tortuous distances, are only weakly correlated, cf. Fig. S16. For the tortuous path, the signal velocity was found to be 0.33 m/s with $R^2 = 0.38$.

Damping coefficient
The rebound effect observed for the advancing water-air interface during Haines jump H9 can be modeled as an exponentially damped harmonic wave,

$$z(t) - \mathrm{z}_0 = A\mathrm{e}^{-\lambda(t-t_0)} \sin(\omega(t - \mathrm{t}_0) + \phi) + z_\infty. \qquad \text{Eq. 1}$$

Here, $z(t)$ is the height as a function of time t, A the initial amplitude, λ the decay rate, ω the angular frequency, φ the phase, and z_∞ the equilibrium height. The time offset, t_0, is set to 15.5 ms and z_0 is the height position at the onset of the jump. The damping ratio is defined as $\zeta = \lambda/\sqrt{\lambda^2 + \omega^2}$. To estimate the damping ratio, we used the height estimates obtained from the average water-air interfacial height from the 14 projections, as shown in Fig. 4 in the main article and here in the inset of Fig. S17. The region of interest is within the rectangular area of the inset and the optimal parameters for the five unknown variables in Eq. 1 obtained through a numerical minimization routine (*fminsearch* in Matlab) is also displayed. The damping ratio is thus estimated to be ζ = 0.67 ± 0.03, consistent with an underdamped system in which inertial effects are present. The uncertainty in the damping ratio was estimated by varying the optimized frequency ± 0.12 s^{-1}, with the implications shown in Fig. S17.

Here we emphasize that the observed oscillations and inertial effects cannot be induced by the syringe pump itself. The periodicity of the damped oscillator was found to be $T = 2\pi/\omega = 4.9$ ms whereas the periodicity of the syringe pump flow rate was 0.13 ms. To the best of our knowledge, these two periods are unrelated. Furthermore, we do not observe any oscillation effects in between the Haines jumps. We thus find it unlikely that any potential syringe pump inaccuracy could explain the observed inertial effects.

Ergodicity
Haines jumps evidently progress at high velocities, which implies that the distance traveled within a particular time interval exceeds the length scale of diffusive mixing. Here, we compute the distance traveled by a Haines jump by summarizing the interfacial

displacements for the 10% fastest displacement vectors obtained in the velocity estimate in Fig. S18 for each timestep. The extent of diffusive mixing Δx in time Δt is computed as $\Delta x = \sqrt{D\Delta t}$ using the self-diffusion coefficient of water, $D = 2.299 \cdot 10^{-5}$ cm^2/s (16). Fig. S18a plot the progression of Haines jumps in a 2D sample system, reproduced from Ref. (17). In Fig. S18b, selected Haines jumps from our study are plotted. The dynamics is clearly super-diffusive, which in Ref. (17) is associated with non-ergodic behavior.

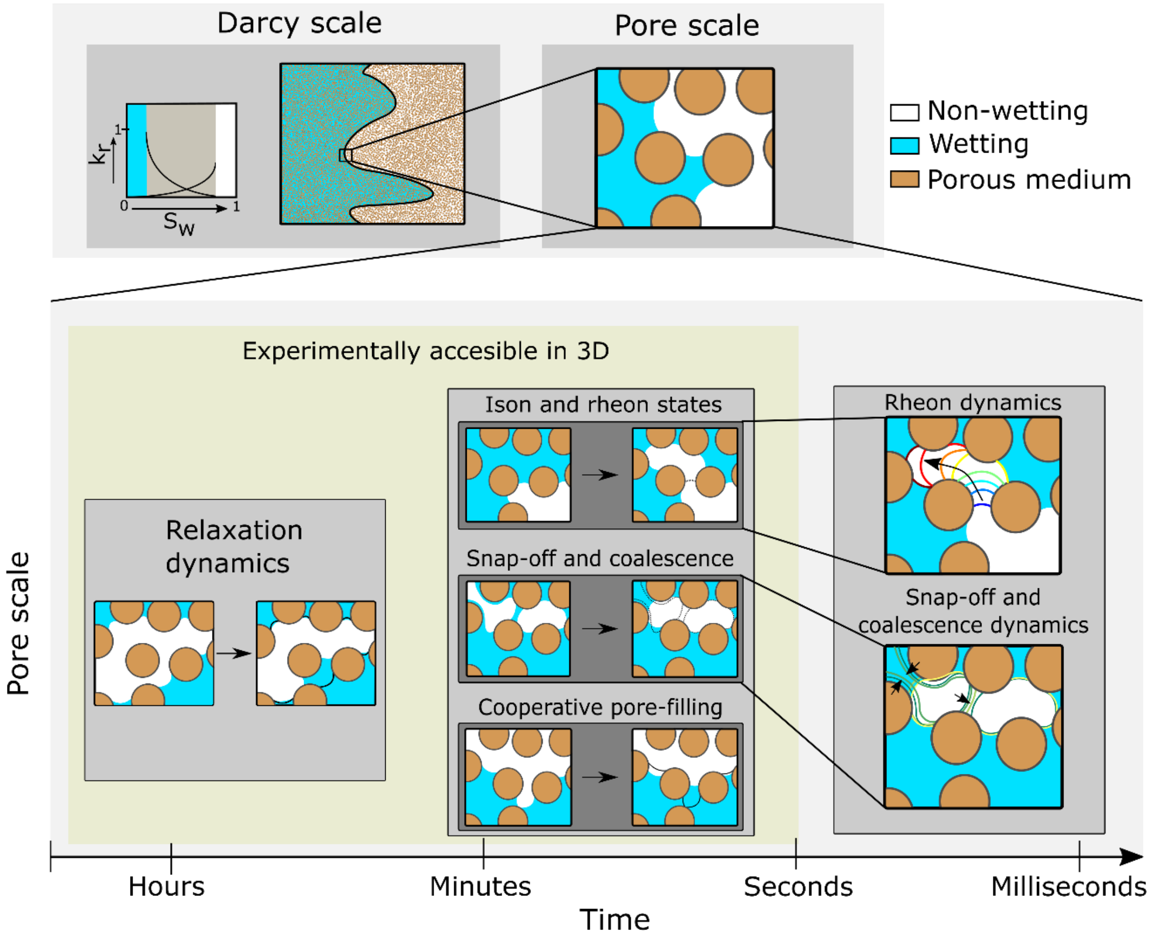

Fig. S1. Spatiotemporal scales of capillarity. Capillary-dominated two-phase flow phenomena span a hierarchy of length- and timescales. At the macroscopic scale, relevant macroscopic properties include saturation, permeability, and capillary pressure. These emergent macroscopic properties are governed by fluid dynamics processes at the pore scale, taking place over multiple timescales. After transient events, slow capillary relaxation dynamics are present. Continuous *ison* interfacial movements change the saturation primarily governed by the injection rate. Phenomena with dynamics in the millisecond range include rheons, piston-like displacements, snap-off, and coalescence.

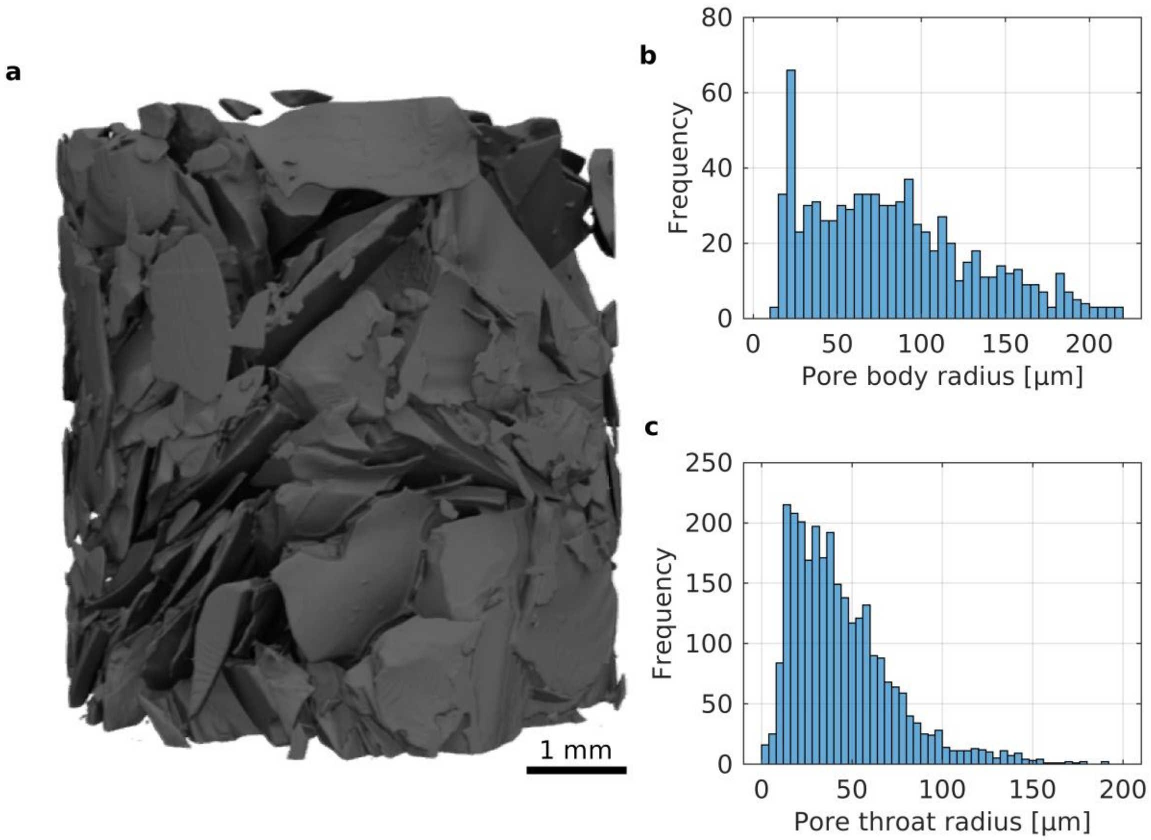

Fig. S2. Static 3D structure of the sintered glass shard sample. Close-up of the porous sample used for the air invasion study. **a** 3D rendering of the glass shards. The initial water saturation has been rendered transparent for clarity. Note the wide distribution of glass shards leaving a variety of pore throat sizes. **b** Pore body radii and **c** pore throat radii. Most of the pore throat radii are around 40 µm, and only a few pore throat radii are larger than 150 µm.

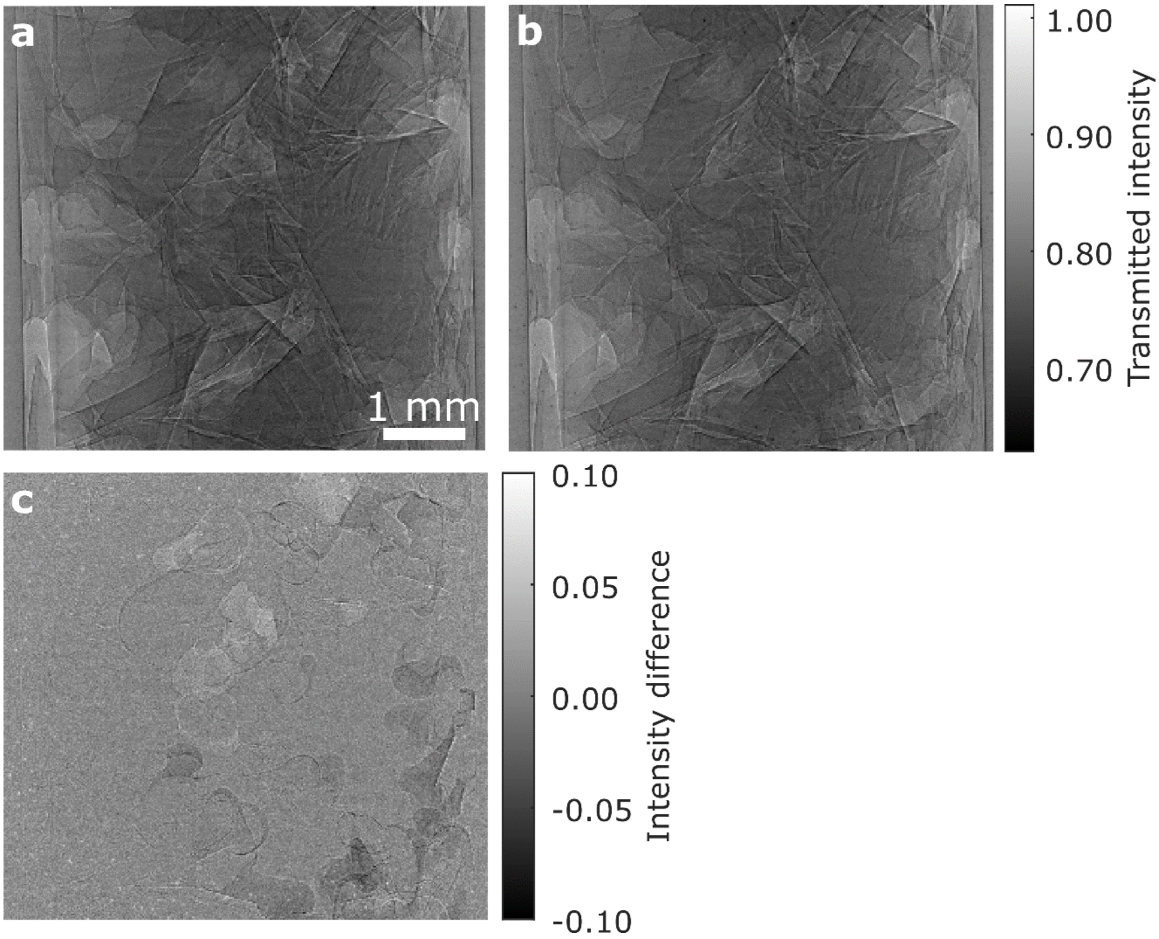

Fig. S3. Check for radiation damage. The 1st and 30th projection series. **a** Radiograph from the 1st projection series and **b** radiograph from the 30th projection series, both obtained at the same tomographic angle. **c** Image difference of **a** and **b** where the intensity bar (arbitrary units) has been modified to better visualize the small differences. Note that all the sharp features from the vertical cylinder walls and the glass shards have vanished, indicating that there were no observable structural changes in the static porous sample matrix during the experiment. The slight residues are caused by slight differences in the saturation as the dynamics were seen to change after the 14th stroboscopic measurement series.

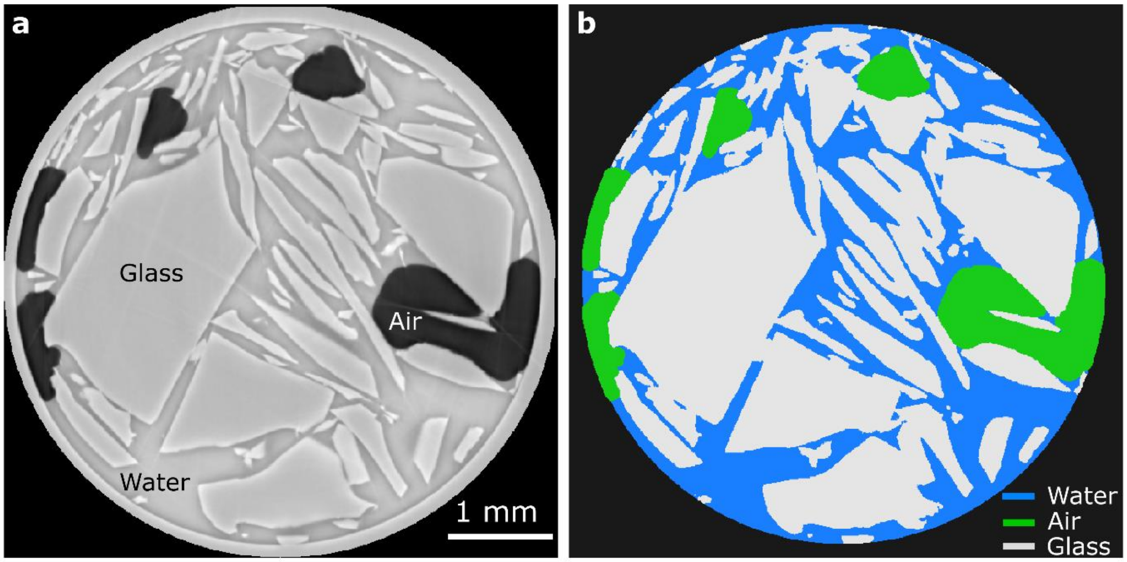

Fig. S4. Segmentation of the high-quality a priori CT data. Scan of the sample and fluids obtained before the fluid cycling. **a** Example of reconstructed tomography slice showing glass shards and doped water in gray values and air in black. **b** Segmented image obtained by a watershed algorithm.

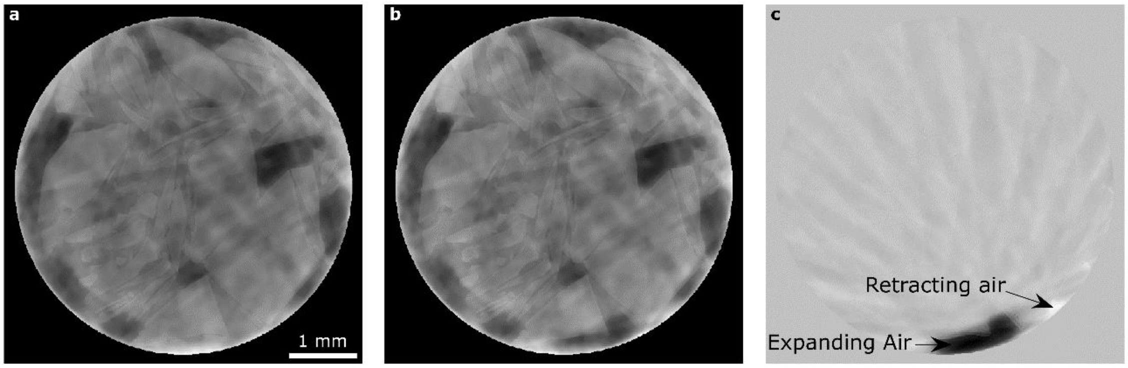

Fig. S5. Difference of volumes highlights dynamics. a Reconstructed slice at time t_0, **b** reconstructed slice at time t_1, and **c** image difference of slices. Although the reconstructions exhibit significant artefacts owing to the few projections, tomogram *differences* can be used to track features that are changing in time. The glass cylinder is not rendered in this figure.

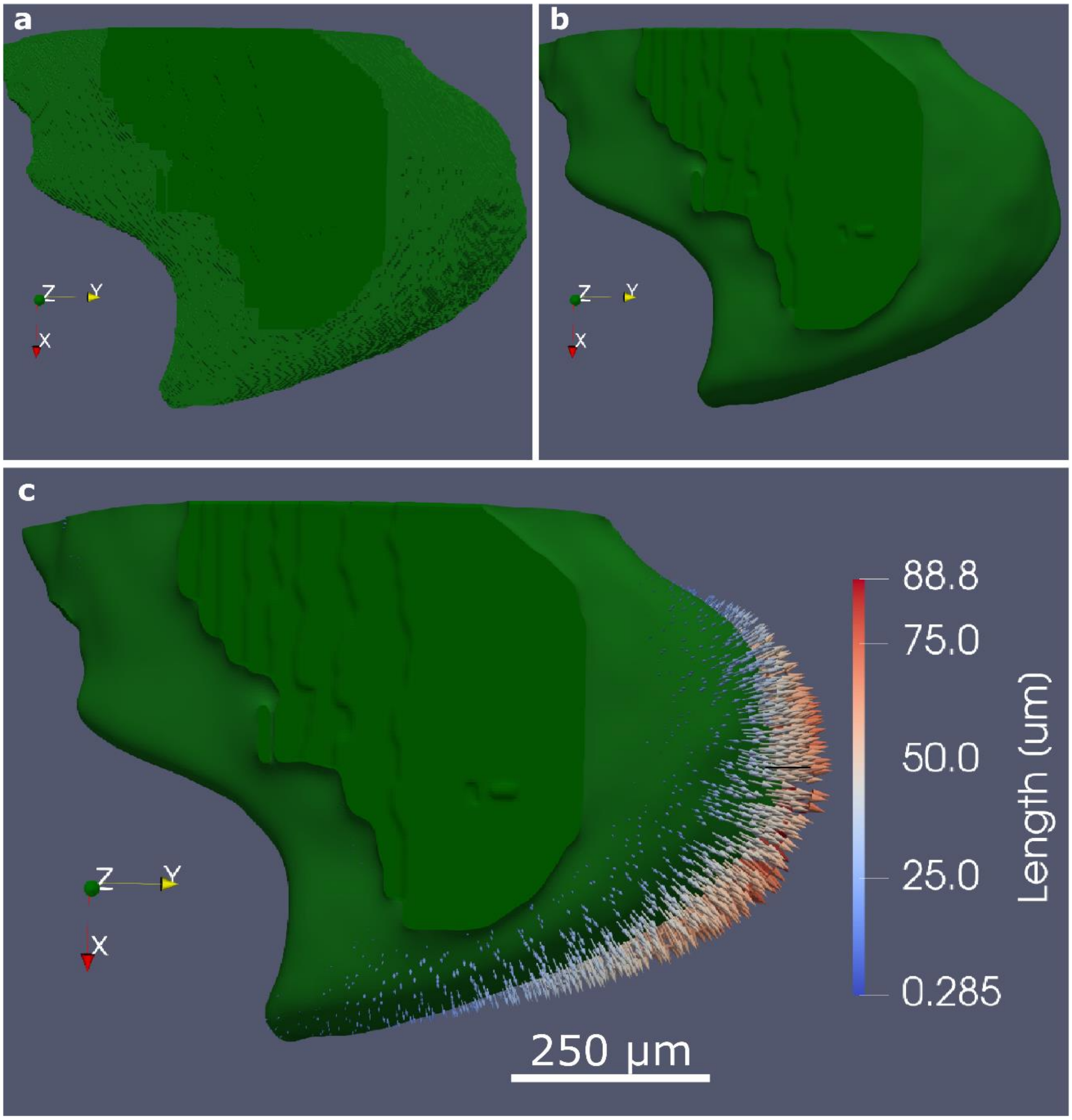

Fig. S6. Estimating the velocity field of the intruding meniscus. a 3D rendering of the volume difference $t_2 - t_1$, where green indicates the moving water-air interface. Here the voxel artefacts are clearly present. **b** Gaussian and curvature smoothed surface. **c** Velocity field of the advancing interface.

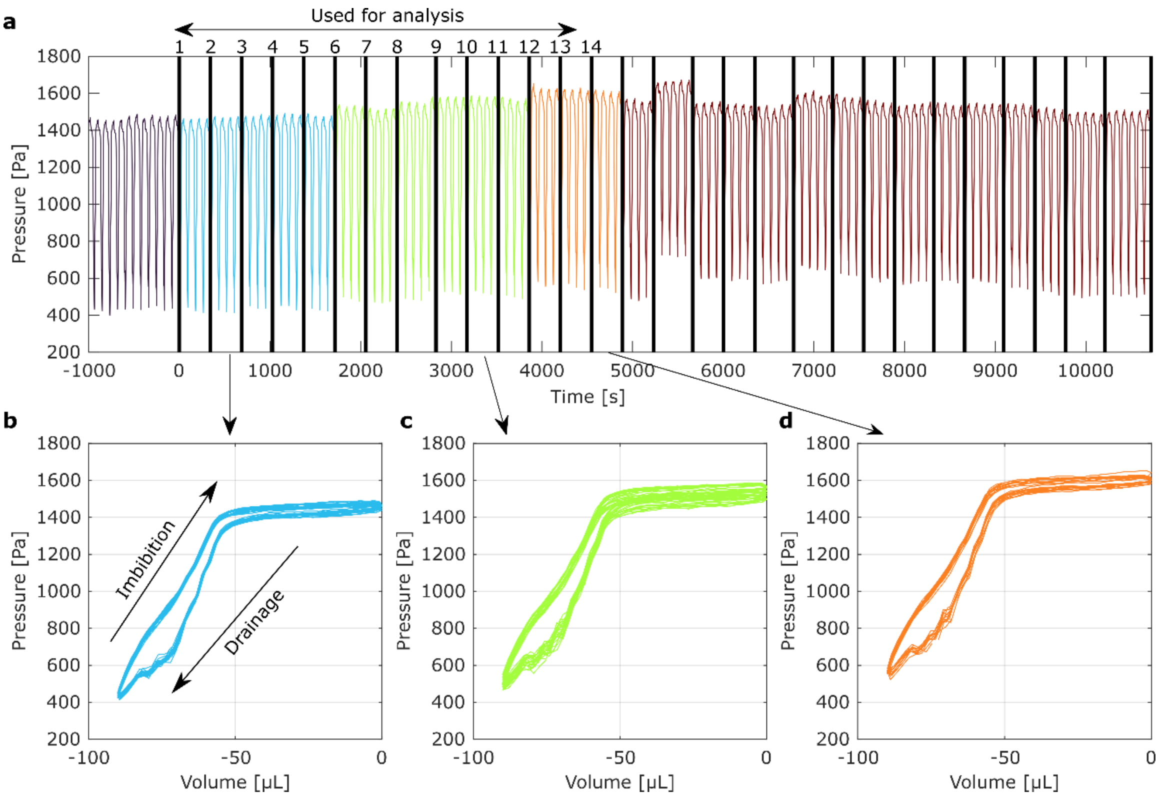

Fig. S7. Pressure history throughout the experiment. a Pressure as a function of time. The black vertical lines indicate the temporal location of the stroboscopic measurements, each lasting approximately 6.5 s. That the step changes coincide with the X-ray irradiation suggests that there is a slight perturbation of the system by the beam. **b-d** Pressure curves as a function of injected/withdrawn volume, clearly showing the hysteretic nature of imbibition and drainage.

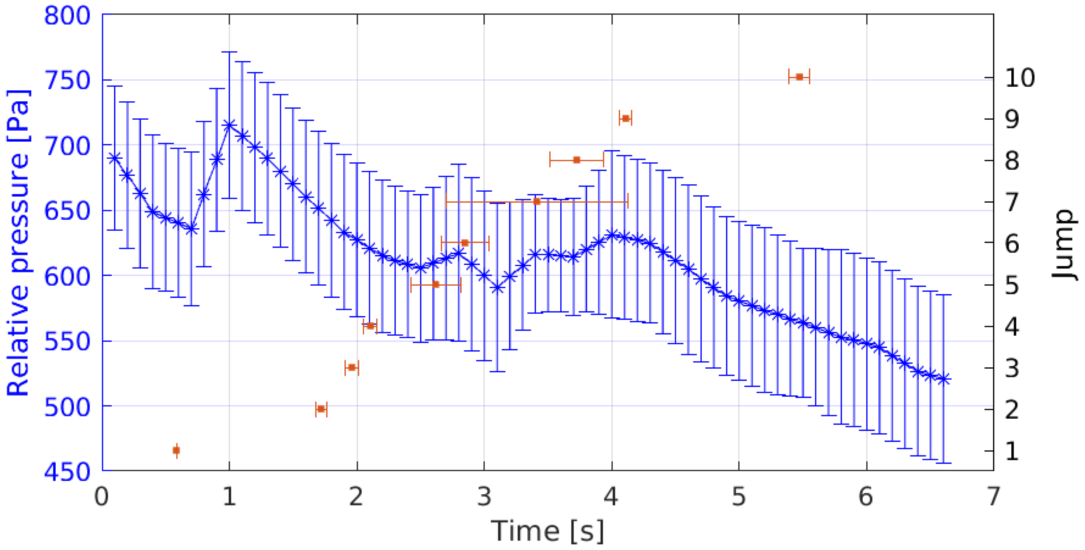

Fig. S8. Pressure readings averaged over the cycles. Here plotted together with the Haines jumps as a function of time during the 6.5 s of radiographic acquisition time. The temporal resolution of the pressure measurements is too low to observe correlations with the Haines jumps. The pressure values are plotted along the left axis with a blue line and the jump index is plotted along the right axis with red symbols.

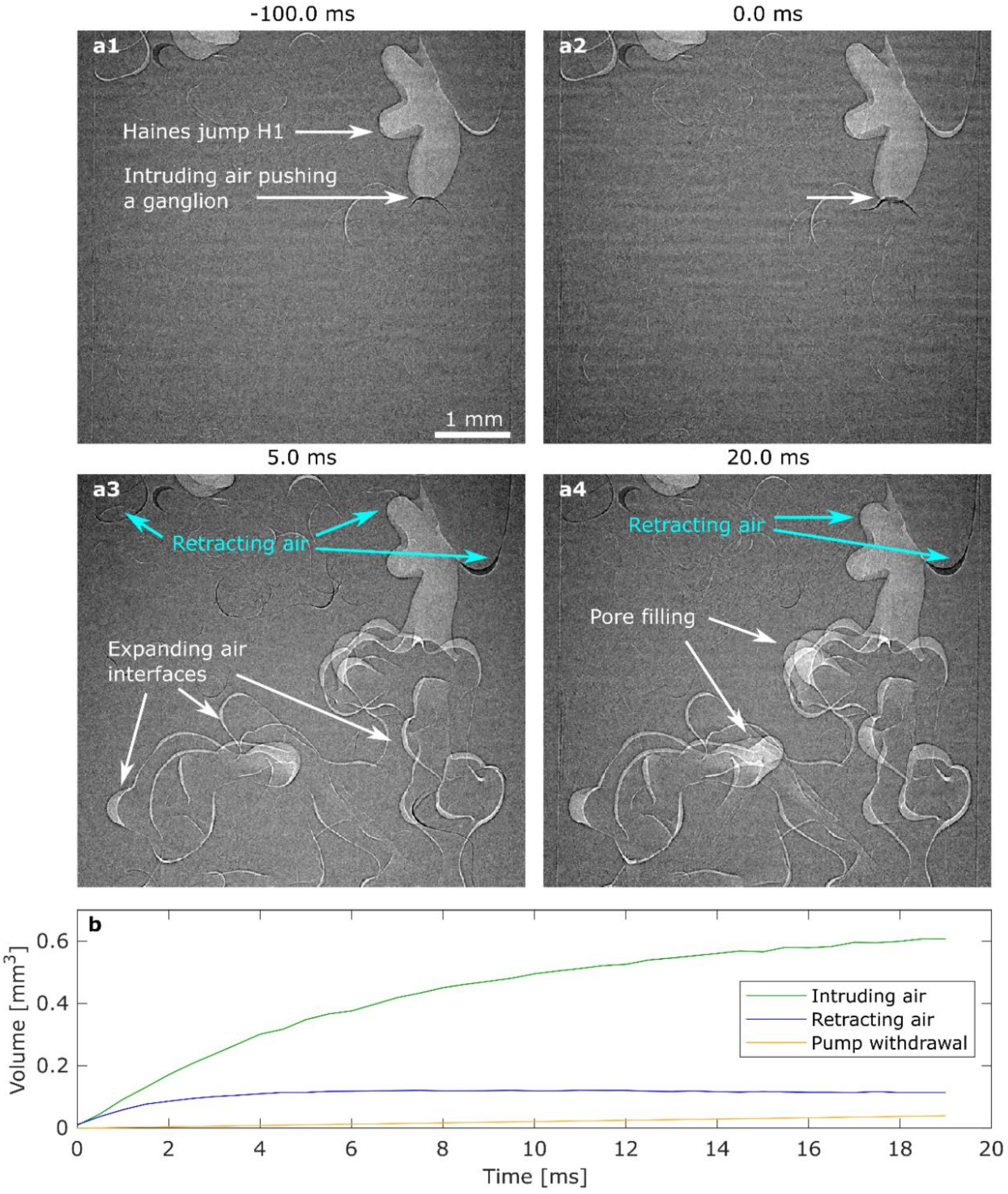

Fig. S9. Coalescence between the intruding air and a ganglion bubble. **a1-a4** Radiographic projections of selected timesteps of the intruding air phase (white regions) pushing the ganglion downwards (dark region in **a1**) and eventually breaks the water layer separating them, causing an avalanche of water-air interfacial movements. In addition to the expansion of air phase, two new pore bodies separated by pore-throats are invaded by the air. A reference frame from the start of the stroboscopic projection series was subtracted from the radiographic images, and the time denotes the onset of the reconnection event. **b** The displaced volume increases steadily and reaches 0.6 mm^3 within 19 ms. The volume changes are computed from the reconstructed tomograms.

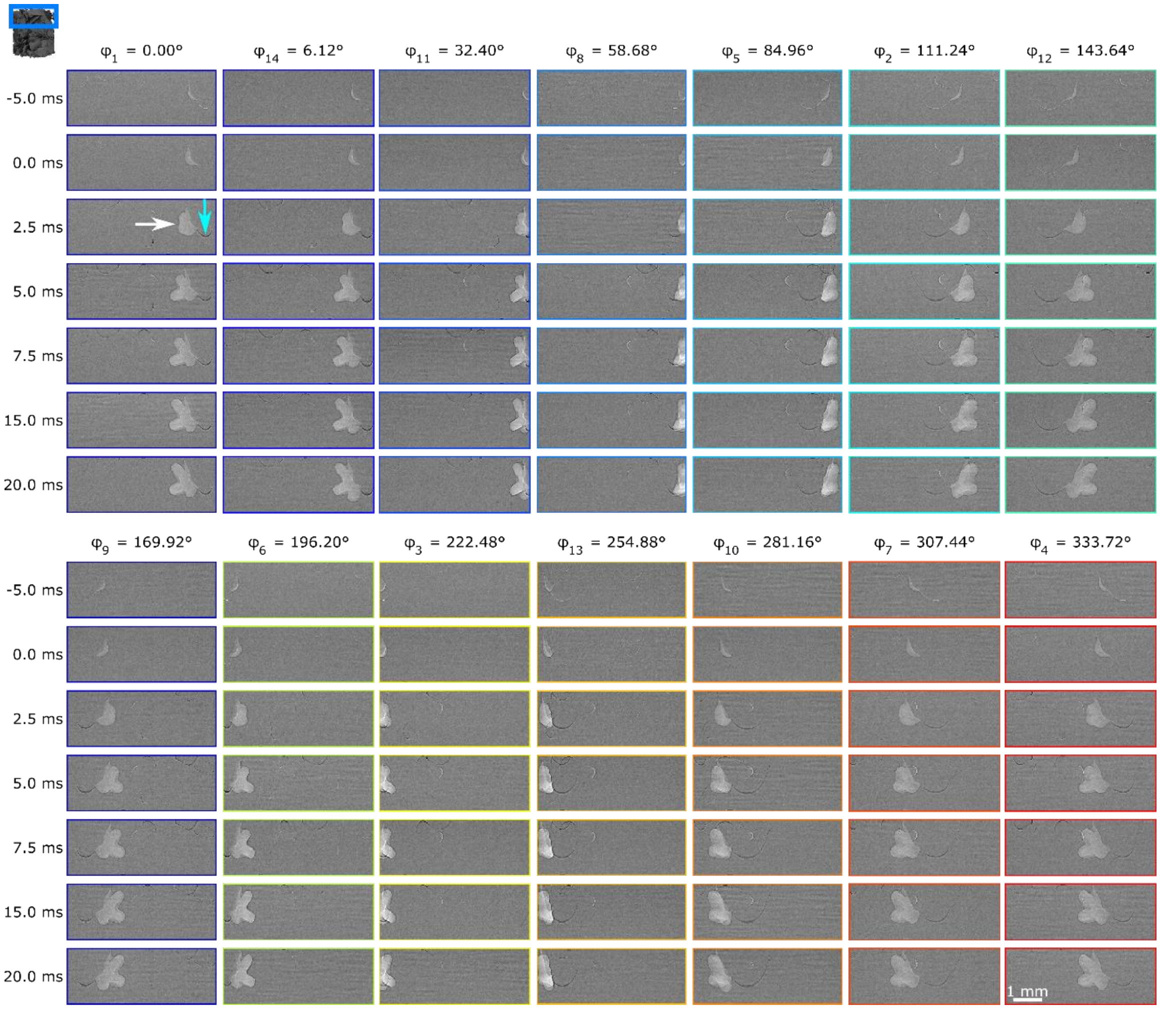

Fig. S10. Stroboscopic projection time series for Haines jump H1. All projection angles (sorted) and a few selected timesteps, are shown. The intruding air phase (white region, marked with white arrow) can be observed to develop into a three-fingered structure, whereas in neighboring regions air can be seen to retract (dark menisci, marked with teal arrow). Reference images obtained 50 ms prior to the onset of the jump have been subtracted to remove the static structures and highlight the dynamics.

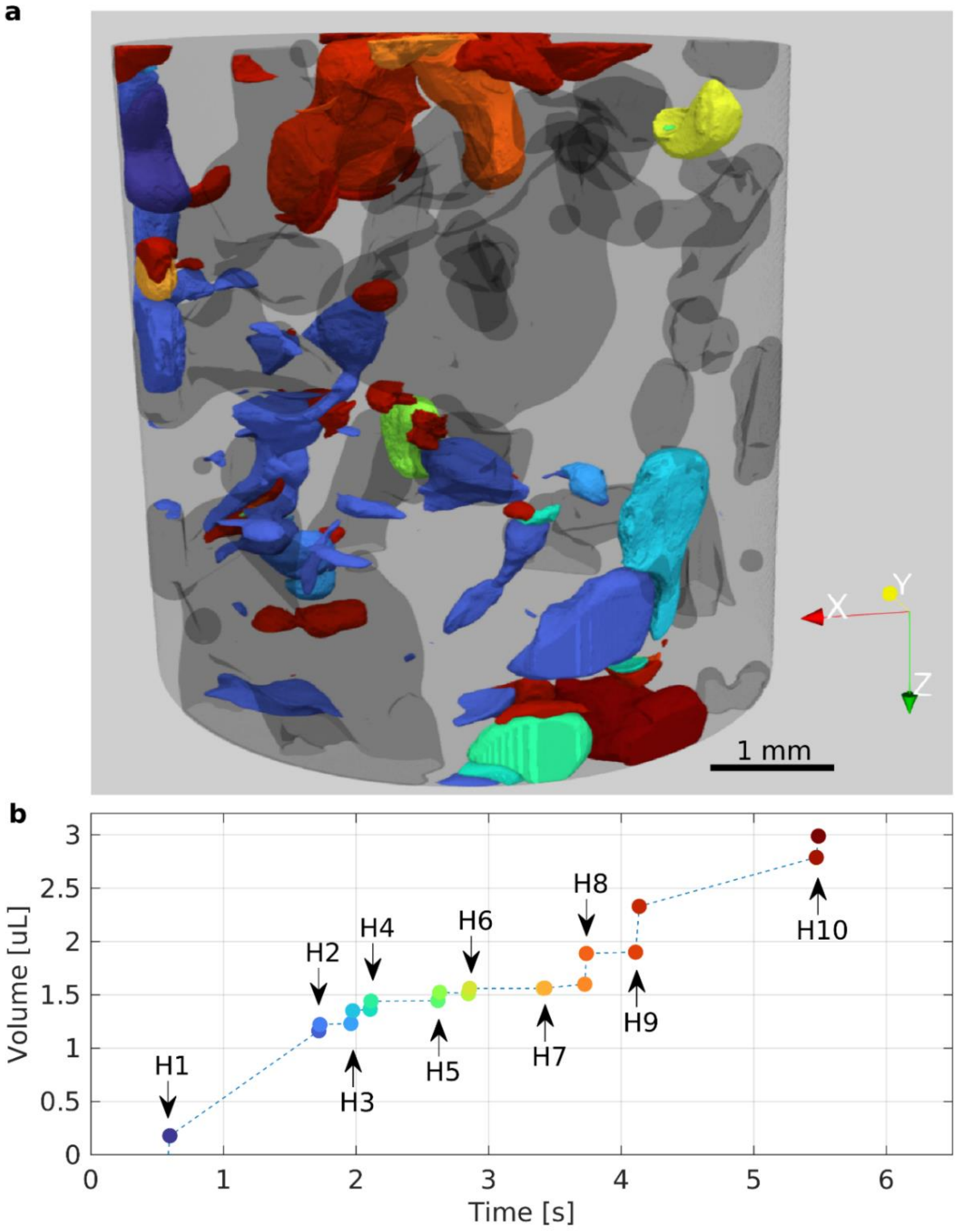

Fig. S11. 4D air invasion dynamics into the porous sample. The color scheme represents the time development, with dark blue denoting early and dark red late developments. **a** Water volume displaced by air from the onset of the first jump to the completion of the last jump. The initial air saturation prior to the start of the dynamics is presented as semi-transparent gray while both glass tube and shards have been omitted for clarity. **b** Displaced water volume as a function of time. The Haines jumps can be seen as sharp changes in volume.

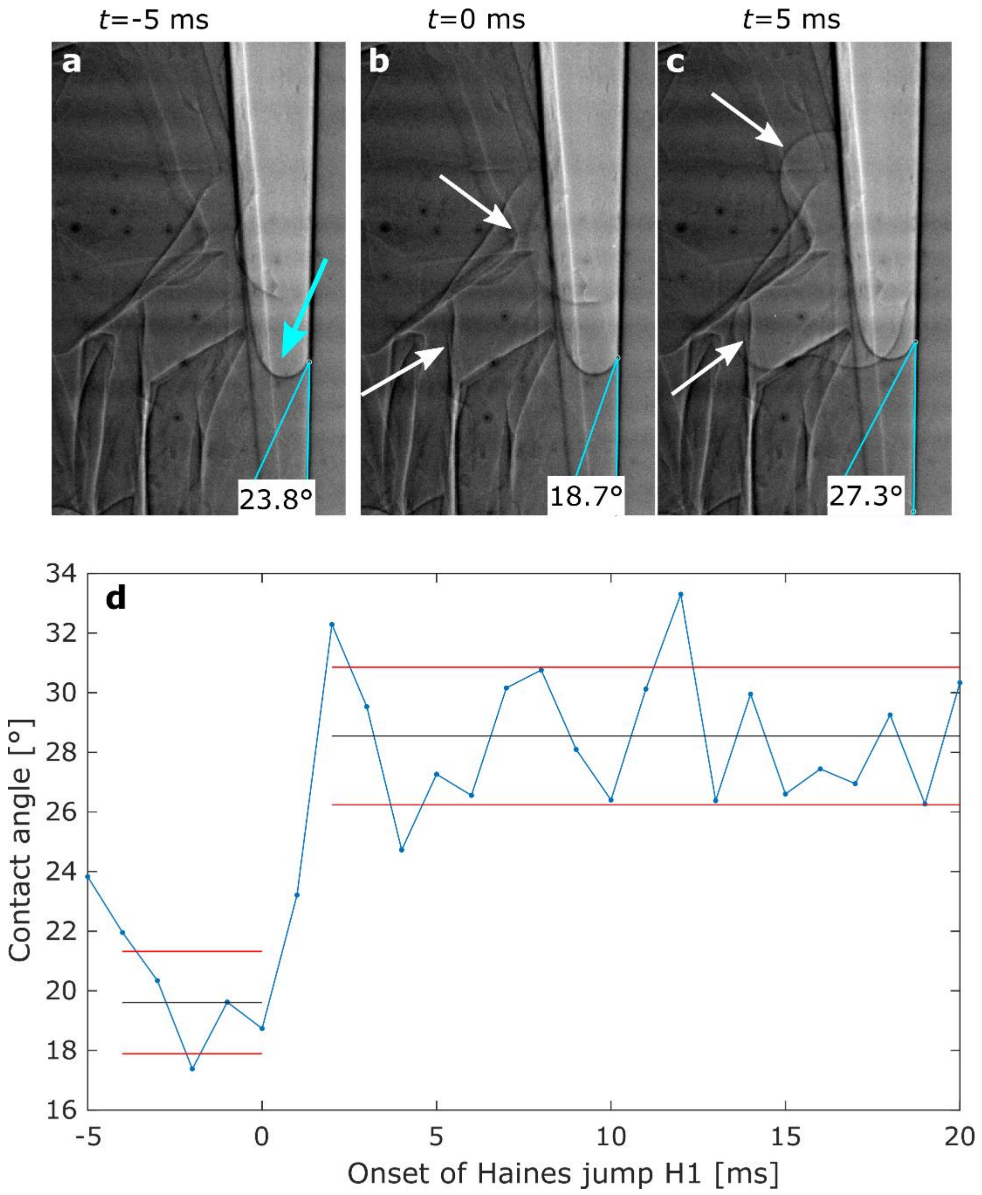

Fig. S12. Estimate of advancing and receding contact angles. Projections before and during Haines jump H1 of a liquid-air front near the H1 event denoted with a teal arrow. **a-c** Illustration of three selected estimates of the contact angle. The vectors liquid-air and liquid-solid are approximately orthogonal to the beam direction. Prior to the jump the measured contact angle corresponds to the receding contact angle, whereas the contact angle during and after the jump approximates the advancing contact angle. The white arrow indicates the intruding air phase during H1 and teal arrow the retracting air phase. **d** The contact angles estimated for 26 time points; 6 points before the jump and 20 points during the jump. A clear distinction before and after the onset of the jump is observed with a mean contact angle of 20° before the jump and 29° during the jump. The black horizontal lines indicate the mean contact angles and the red lines the ± 1 standard deviation.

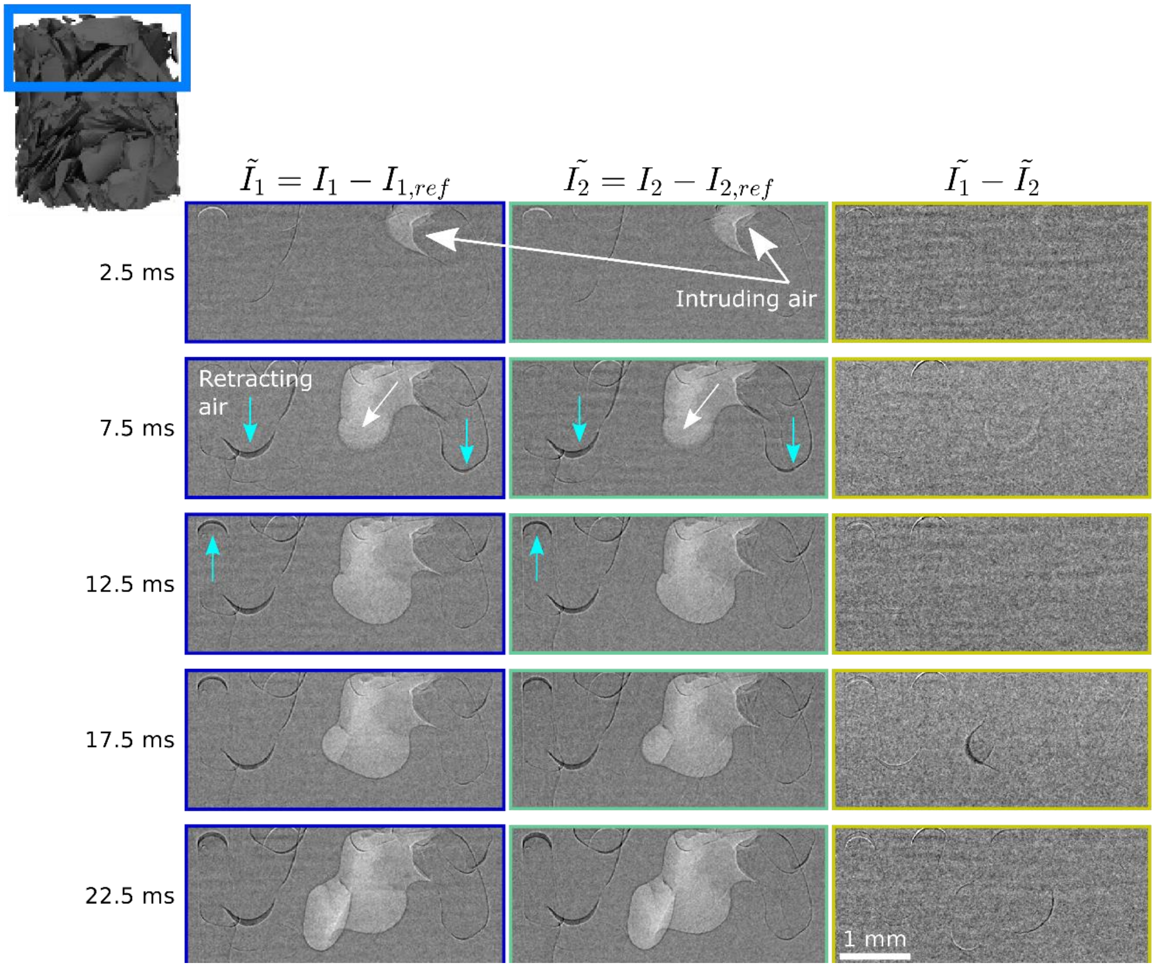

Fig. S13. Repeatability of Haines jump H9. Columns 1 and 2 show the instability as it progresses for two consecutively measured stroboscopic projection series obtained at the same angular orientation of the sample, where bright gray indicates intruding air and darker regions indicate retracting air. A reference image obtained 50 ms prior to the onset of the jump has been subtracted to better highlight the dynamics. The third column shows the difference of columns 1 and 2, with the nearly featureless appearance testifying to the repeatability of the Haines jump.

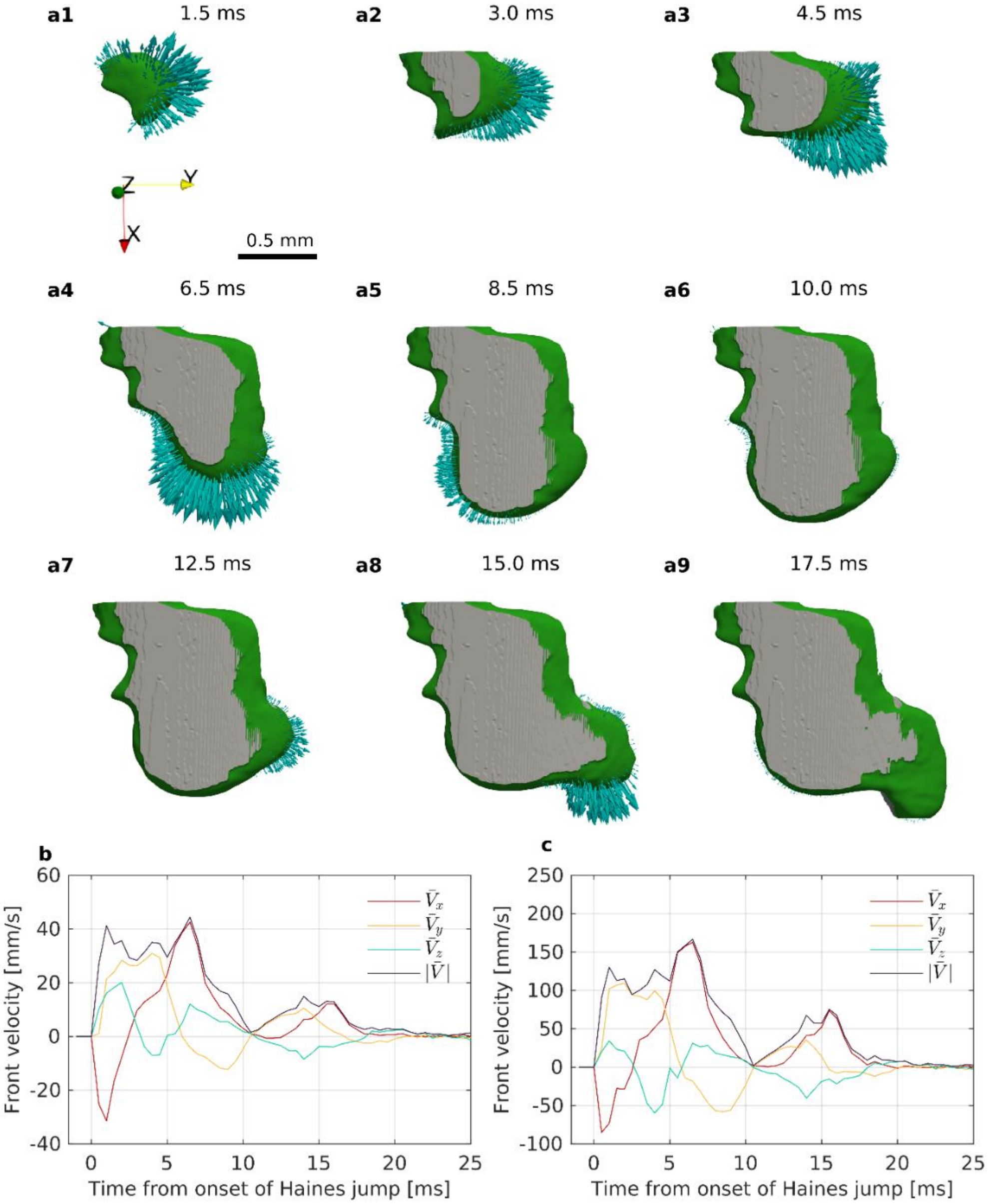

Fig. S14. Interfacial velocity fields for the advancing air-water meniscus. Data for Haines jump H9. **a** Interfacial velocity field superposed on the air-water meniscus during the Haines jump at selected timesteps. Air-water and air-solid interfaces are colored in green and gray, respectively. **b** Average velocity values for three orthogonal directions as well as the average total velocity. **c** Average velocity profiles of the 10% largest velocity vectors, providing insight into the interfacial regions that move the most rapidly. Note that the velocity field is zero prior to the jump and that the field returns to a full standstill when the jump has finished.

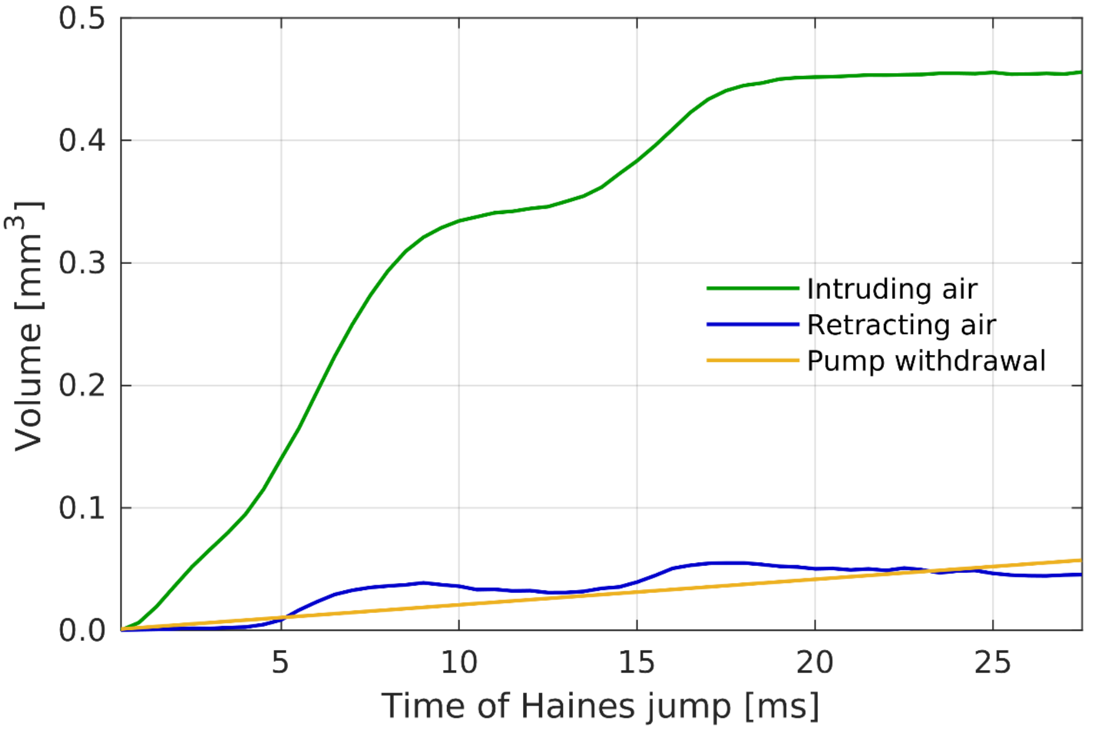

Fig. S15. Volume balance during Haines jump H9. The intruding and retracting air volumes were estimated from the CT reconstructions, while the (water) volume withdrawn by the pump was estimated from the syringe pump specifications. The change in volume reached a plateau at 11 ms and stabilized after 20 ms. Plausible reasons for the non-constant saturation within the FoV are discussed in SI Appendix 2.

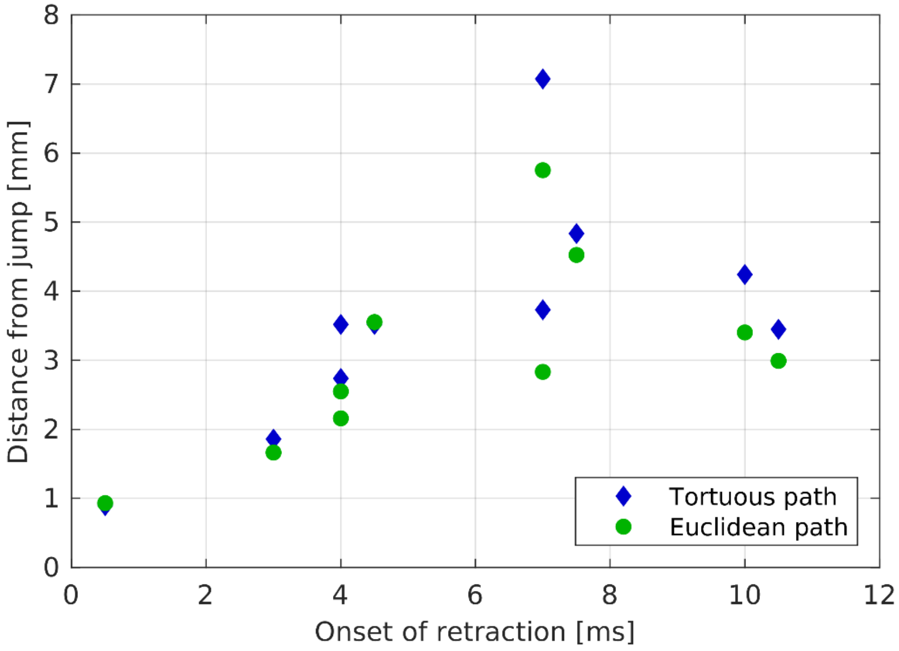

Fig. S16. Instability delay across zone of influence. The distance retracting air volumes were located from the Haines jump H9, plotted as a function of time, measured along both Euclidean and tortuous paths. While there is significant correlation in the close vicinity of the jump, there is essentially no linear correlation at the largest separation distances.

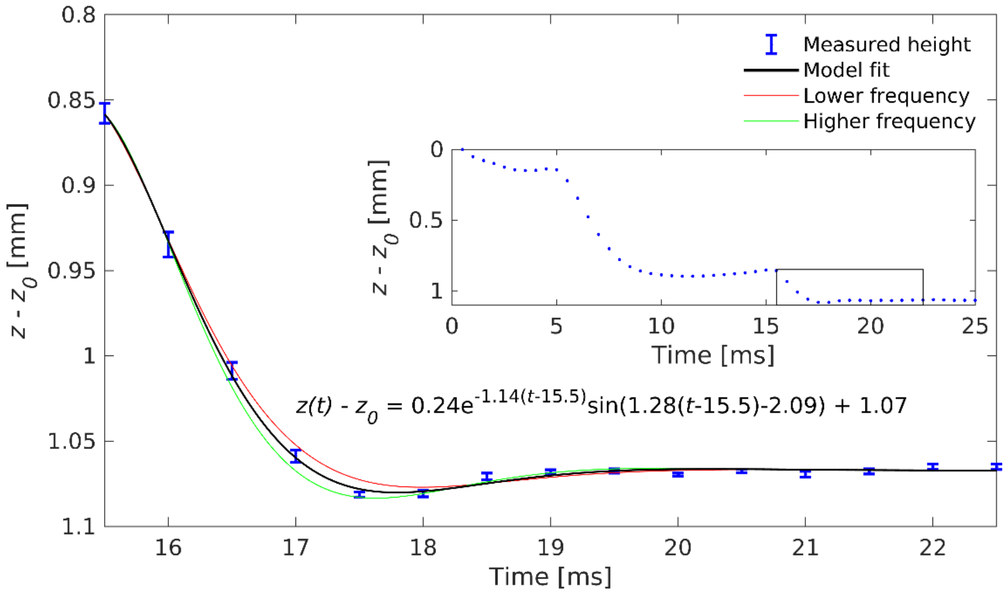

Fig. S17. Mathematical fit of meniscus rebound oscillations. The inset shows the average projection height z for the 14 projections obtained for the region of interest shown in the inset with the error bars denoting one standard deviation. To give an impression of the uncertainty, model perturbations with angular frequency shifts of +0.12 $\mathbf{s^{-1}}$ (–0.12 $\mathbf{s^{-1}}$) are shown in green (red).

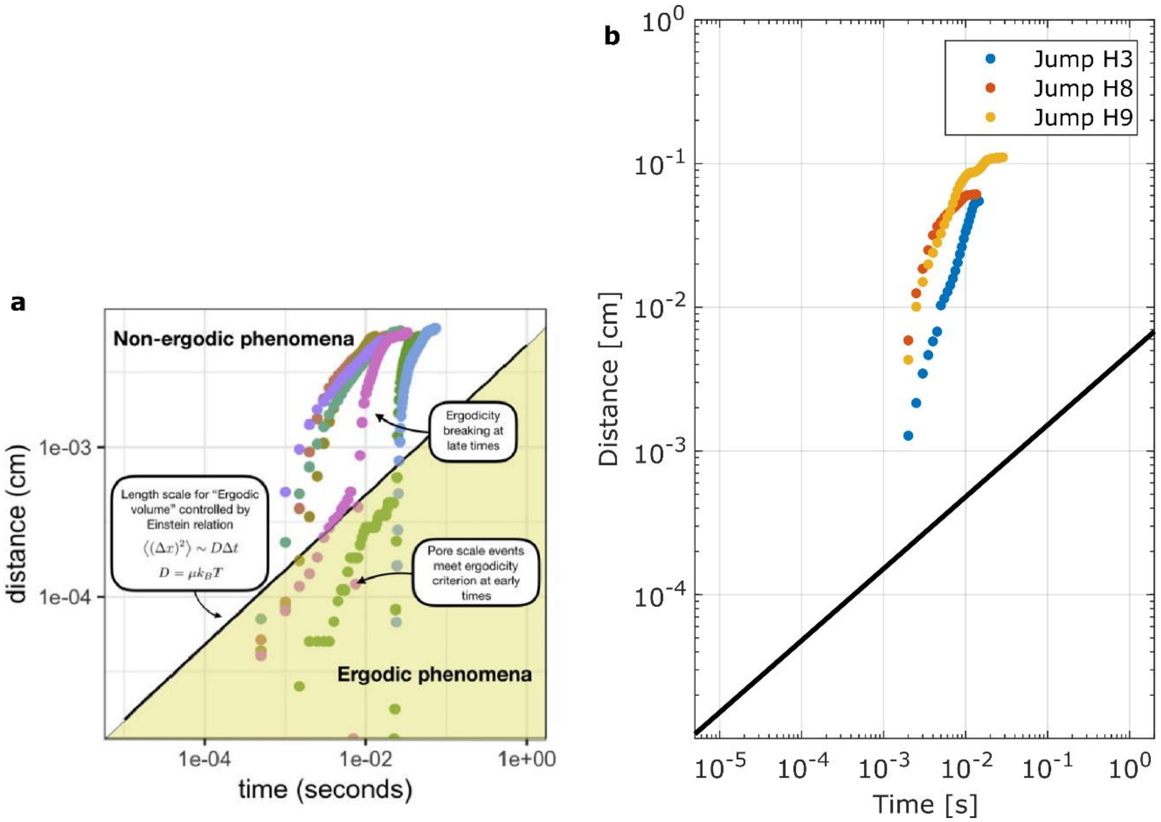

Fig. S18. Displacement as function of time during Haines jumps. a Haines jumps as observed in a 2D micro-model (17). **b** In our study, the Haines jumps are as expected observed to be super-diffusive, here exemplified with H3, H8 and H9. In (17) such behavior is associated with non-ergodicity. Subfigure **a** is reproduced from (17) with admission from the American Institute of Physics.

Movie S1 (separate file). Visualization of the first stroboscopic radiographic projection series containing a total of 13000 images. The first image has been subtracted from the consecutive images in order to visualize the intruding (white regions) and retracting (darker regions) air phase.

Movie S2 (separate file). Visualization of the displaced air occurring over a reconstructed time of 5.6 s with a temporal resolution of 200 ms. Green indicates the intruding air phase, whereas the gray phase indicates the initial air distribution.

Movie S3 (separate file). Visualization of the interfacial dynamics during Haines jump H9 in the main text. Note how the intruding air is redirected three times throughout the dynamics and how it bounces back in the end.

SI References

1. J. R. Rumble, *CRC Handbook of Chemistry and Physics* (2014) https:/doi.org/10.1201/b17118.
2. K. Ali, A. ul H. A. Shah, S. Bilal, A. ul H. A. Shah, Surface tensions and thermodynamic parameters of surface formation of aqueous salt solutions: III. Aqueous solution of KCl, KBr and KI. *Colloids Surfaces A Physicochem. Eng. Asp.* **337**, 194–199 (2009).
3. Z. Sun, J. C. Santamarina, Haines jumps: Pore scale mechanisms. *Phys. Rev. E* **100** (2019).
4. R. Lenormand, E. Touboul, C. Zarcone, Numerical models and experiments on immiscible displacements in porous media. *J. Fluid Mech.* **189**, 165–187 (1988).
5. Y. Méheust, G. Løvoll, K. J. Måløy, J. Schmittbuhl, Interface scaling in a two-dimensional porous medium under combined viscous, gravity, and capillary effects. *Phys. Rev. E* **66**, 051603 (2002).
6. T. Köhler, A projection access scheme for iterative reconstruction based on the golden section in *IEEE Symposium Conference Record Nuclear Science 2004.*, (2004), pp. 3961–3965.
7. A. Serrano, F. Gálvez, O. R. De La Fuente, M. A. García, X-ray irradiation of soda-lime glasses studied in situ with surface plasmon resonance spectroscopy. *J. Appl. Phys.* **113**, 113104 (2013).
8. M. Rajaram, E. J. Friebele, Effects of radiation on the properties of low thermal expansion coefficient materials: A review. *J. Non. Cryst. Solids* **108**, 1–17 (1989).
9. D. C. F. Monteiro, *et al.*, A microfluidic flow-focusing device for low sample consumption serial synchrotron crystallography experiments in liquid flow. *J. Synchrotron Radiat.* **26**, 406–412 (2019).
10. R. P. Xian, *et al.*, A multiscale X-ray phase-contrast tomography dataset of a whole human left lung. *Sci. Data* **9** (2022).
11. B. M. Weon, J. S. Lee, J. H. Je, K. Fezzaa, X-ray-induced water vaporization. *Phys. Rev. E* **84**, 032601 (2011).
12. S. M. Walker, *et al.*, In Vivo Time-Resolved Microtomography Reveals the Mechanics of the Blowfly Flight Motor. *PLoS Biol.* **12**, 1001823 (2014).
13. R. Mokso, *et al.*, Four-dimensional in vivo X-ray microscopy with projection-guided gating. *Sci. Reports 2015 51* **5**, 1–6 (2015).
14. A. AlRatrout, A. Q. Raeini, B. Bijeljic, M. J. Blunt, Automatic measurement of contact angle in pore-space images. *Adv. Water Resour.* **109**, 158–169 (2017).
15. M. Tuller, D. Or, Hydraulic conductivity of variably saturated porous media: Film and corner flow in angular pore space. *Water Resour. Res.* **37**, 1257–1276 (2001).
16. M. Holz, S. R. Heil, A. Sacco, Temperature-dependent self-diffusion coefficients of water and six selected molecular liquids for calibration in accurate 1H NMR PFG measurements. *Phys. Chem. Chem. Phys.* **2**, 4740–4742 (2000).
17. J. E. McClure, S. Berg, R. T. Armstrong, Capillary fluctuations and energy dynamics for flow in porous media. *Phys. Fluids* **33**, 83323 (2021).
